# Nephrotoxicity evaluation and proteomic analysis in kidneys of rats exposed to thioacetamide

**DOI:** 10.1038/s41598-022-11011-3

**Published:** 2022-04-27

**Authors:** Ji-youn Lim, Woon-Won Jung, Woojin Kim, Kyoung-Sik Moon, Donggeun Sul

**Affiliations:** 1 Graduate School of Medicine, Korea University, Seoul, 136-705 Republic of Korea; 2 Department of Biomedical Laboratory Science, College of Health Science, Cheongju University, Cheongju, 28503 Republic of Korea; 3 Department of Advanced Toxicology, Korea Institute of Toxicology, 141 Gajeong-ro, Yuseong-Ku, Daejeon, 34114 Republic of Korea; 4 Present Address: New Drug Development Center, Daegu-Gyeongbuk Medical Innovation Foundation (DGMIF), Daegu, 41061 Republic of Korea

**Keywords:** Biochemistry, Biotechnology

## Abstract

Thioacetamide (TAA) was administered orally at 0, 10, and 30 mg/kg body weight (BW) daily to Sprague–Dawley rats aged 6–7 weeks for 28 consecutive days. Nephrotoxicity and proteomics were evaluated in the kidneys of rats exposed to TAA. The BW decreased, however, the relative kidneys weight increased. No significant histopathologic abnormalities were found in the kidneys. The numbers of monocytes and platelets were significantly increased. However, the mean corpuscular volume and hematocrit values were decreased significantly in rats exposed to 30 mg/kg BW TAA. The expression levels of Kim-1 and NGAL were increased 4 to 5-fold in the kidneys, resulting in significant nephrotoxicity. Proteomic analysis was conducted and a total of 5221 proteins spots were resolved. Of these, 3 and 21 protein spots were up- and downregulated, respectively. The validation of seven proteins was performed by Western blot analysis. The expression level of ASAP2 was significantly upregulated, whereas RGS14, MAP7Dl, IL-3Rα, Tmod1, NQO2, and MUP were reduced. Sixteen isoforms of MUP were found by the 2DE immunoblot assay and were significantly downregulated with increasing exposure to TAA. MUP isoforms were compared in the liver, kidneys, and urine of untreated rats and a total of 43 isoforms were found.

## Introduction

Thioacetamide (TAA) is a carcinogen and hepatotoxicant that has been used for the experimental induction of hepatic damage in numerous animals including rats and mice^1–6^. In addition, TAA is also known as a nephrotoxicant^7–15^. In previous research, TAA induced changes in trace elements and structural kidney damage, which increased collagen deposition in the renal medulla and fibrin in the tubules^8^. TAA also caused the death of cells in the terminal portion of the proximal renal tubules^7^. TAA administration resulted in impaired renal functions including severe tubular epithelial cell death associated with inflammatory cell infiltration and glomerular congestion^9^. Furthermore, TAA showed the severe renal tissue infiltration of inflammatory cells, degeneration, sclerosis and necrosis of the glomeruli, interstitial fibrosis, dilated tubules with necrotic tubular cells, and epithelial shedding^11,13–15^.

Toxico-proteomic analysis is a method employed for analyzing differential gene expression at the protein level and identifying critical proteins by comparing the proteomic patterns under different conditions after exposure to toxicological compounds^16–20^.

For the proteomic study, we chose two concentrations of TAA (10 and 30 mg/kg BW), which did not induce significant pathological damages, but induced nephrotoxicity in the kidneys. These results were based on histopathological analysis and Western blot assays of kidney injury biomarkers. In addition, we also determined the hematological toxicity in rats exposed to two low doses of TAA. Proteomic analysis was performed using three different isoelectric point (pI) ranges (3–5.6, 4–7, and 6–9) and large-size two-dimensional gel electrophoresis. Mass spectrometry and Western blot analyses were applied to identify and validate toxicological biomarkers of TAA in the kidneys. Ultra-performance liquid chromatography-electrospray ionization-mass spectrometry (UPLC-ESI-MS) analysis was applied to identify the proteins. Some proteins showed up- and downregulation and their expression levels were confirmed by Western blot analysis. Among them, several major urinary proteins (MUPs) were significantly downregulated, and two-dimensional immunoblot analysis was performed to identify the MUP isoforms and determine their expression. MUP isoform profiles were compared in the liver, kidneys, and urine of untreated rats.

## Results

### Determination of body weight and relative organ weight to body weight of rats exposed to TAA

The body weight of the TAA-treated groups decreased with increasing doses of TAA compared to the control group. The body weight of rats exposed to 30 mg/kg BW TAA was significantly decreased compared to the control group (*p* < 0.001). The relative organ weight to body weight was measured in the kidneys of rats exposed to 10 and 30 mg/kg BW TAA. The relative liver and kidney weights were significantly increased in rats exposed to 30 mg/kg BW of TAA compared to the control group (liver and kidney: *p* < 0.001 and *p* < 0.05, respectively) (Table 1).

**Table 1:** Relative organ weight to body weight of rats exposed to TAA.

	Thioacetamide (mg/kg BW)		
	0	10	30
Body weight (g)	351.3 ± 9.30	345.1 ± 32.11	288.3 ± 13.81***
Relative kidney weight (%)	0.87 ± 0.04	0.85 ± 0.05	0.95 ± 0.06*

Significant changes were indicated with *p < 0.05 and ***p < 0.001.

### Hematological and histopathological analysis

**Figure 1:**
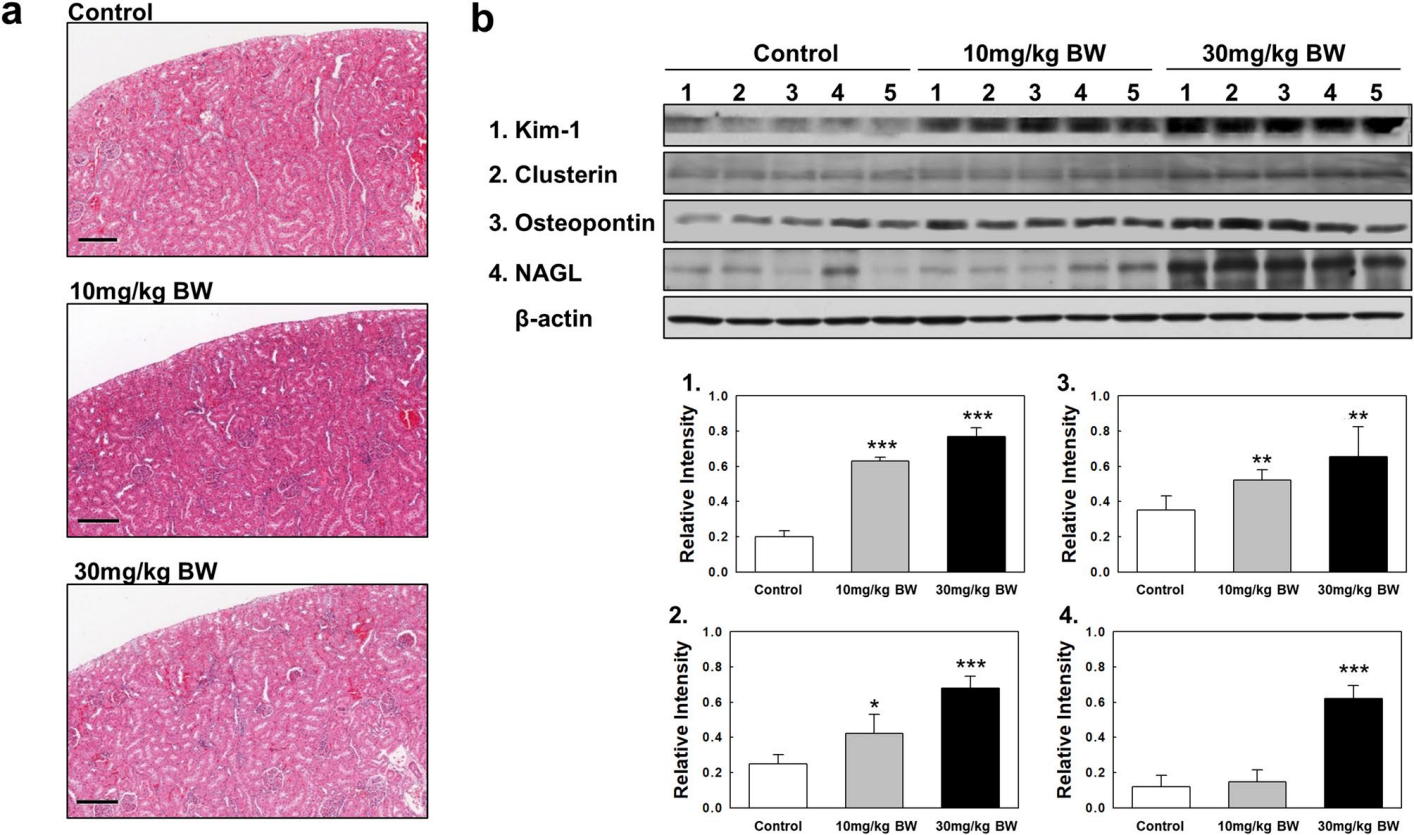
Histopathological observations and determination of kidney injury biomarkers using Western blot analysis. (**a**) Histopathological observations in kidneys. Kidney tissues were fixed in 10% formalin and then embedded in paraffin. Tissue slides of 5-μm-thick sections were stained with hematoxylin and eosin and then observed by light microscopy (scale bar = 200 μm; magnification, × 100). Control group (0.5% carboxymethyl cellulose as vehicle only), 10 mg/kg BW TAA, 30 mg/kg BW TAA. (**b**) Western blot analysis of Kim-1, NGAL, osteopontin, and clusterin proteins in rat kidney tissue. (1) Kim-1, (2) NGAL, (3) osteopontin, and (4) clusterin. Original blots are presented in Supplementary Western blot data. Quantities represented by the gel bands are expressed as intensity relative to β-actin. All relative intensity results are presented as the means ± SD of five experiments. *, **, and *** indicate *p*-values of 0.05, 0.01 and 0.001, respectively compared to the control.

Table 2 shows the hematological changes in rats exposed to 10 and 30 mg/kg BW TAA. There was no significant change in total white blood cell (WBC) counts in rats exposed to 10 and 30 mg/kg BW TAA compared to the control group (Table 2). However, the numbers of monocyte (MO) and platelet (PLT) were significantly increased in rats exposed only to 30 mg/kg BW TAA compared to the control group (*p* < 0.001 and *p* < 0.05, respectively) (Table 2). In erythrocytic parameters, the mean corpuscular volume (MCV) and hematocrit (HCT) values were significantly decreased in rats exposed to 30 mg/kg BW TAA compared to the control group (*p* < 0.01). Histopathological analysis was performed to evaluate pathological toxic effects in the kidneys of rats exposed to TAA. Histopathological observations of basophilia, casts, cysts, inflammatory cell foci, and interstitial fibrosis in the kidney tissue were performed. A very slight degree of basophilia was found (Supplementary Fig. S1 and Table S1), but no significant histopathologic abnormalities were revealed in the kidneys of rats exposed to TAA (Fig. 1A and Table S1).

**Table 2:** Hematological values of rats exposed to TAA.

Parameters	Thioacetamide ( mg/kg BW)		
	0	10	30
WBC (× 10^3^/uL)	9.30 ± 2.60	10.55 ± 1.44	11.37 ± 1.52
LY (× 10^3^/uL)	7.65 ± 2.33	8.98 ± 1.24	8.74 ± 1.15
MO (× 10^3^/uL)	0.23 ± 0.10	0.28 ± 0.07	0.47 ± 1.15***
EO (× 10^3^/uL)	0.06 ± 0.04	0.08 ± 0.02	0.08 ± 0.06
BA (× 10^3^/uL)	0.05 ± 0.02	0.05 ± 0.01	0.04 ± 0.02
PLT (× 10^3^/uL)	1173.2 ± 69.26	1211.8 ± 55.35	1379.4 ± 191.53*
RBC (× 10^6^/uL)	8.60 ± 0.49	8.63 ± 0.73	8.24 ± 0.33
HCT (%)	51.72 ± 2.45	50.64 ± 4.00	46.94 ± 0.87**
MCV (fL)	60.14 ± 1.04	58.70 ± 1.54	56.96 ± 1.52**
MCH (pg)	19.10 ± 0.33	18.82 ± 0.36	18.44 ± 0.60
MCHC (g/dL)	31.78 ± 0.38	32.06 ± 0.53	32.36 ± 0.30

*WBC* white blood cells, *LY* lymphocytes, *MO* monocytes, *EO* eosinophils, *BA* basophils, *PLT* platelets, *RBC* red blood cells, *HCT* hematocrit, *MCV* mean corpuscular volume, *MCH* mean corpuscular hemoglobin, *MCHC* mean corpuscular hemoglobin concentration.

Significant changes were indicated with *p < 0.05, **p < 0.01, and ***p < 0.001.

### Determination of kidney injury biomarkers using Western blot analysis

Four kidney injury biomarkers including kidney injury molecule-1 (Kim-1), neutrophil gelatinase-associated lipocalin (NGAL), osteopontin, and clusterin were analyzed by Western blots to evaluate nephrotoxicity. The expression levels of Kim-1 and NGAL were increased four to fivefold in the kidney tissue of rats exposed to the high concentration of TAA compared to the control group (Fig. 1B). Furthermore, the expression of two kidney injury biomarkers Kim-1 and NGAL was evaluated in the urine of the rats. Their expression was very significantly increased in the urine of rats exposed to TAA compared to the control group (Supplementary Fig. S2). Original blots are presented in Supplementary Western blot data.

### 2-DE analysis of expressed proteins in a dose-dependent manner by kidney tissue exposed to TAA

**Figure 2:**
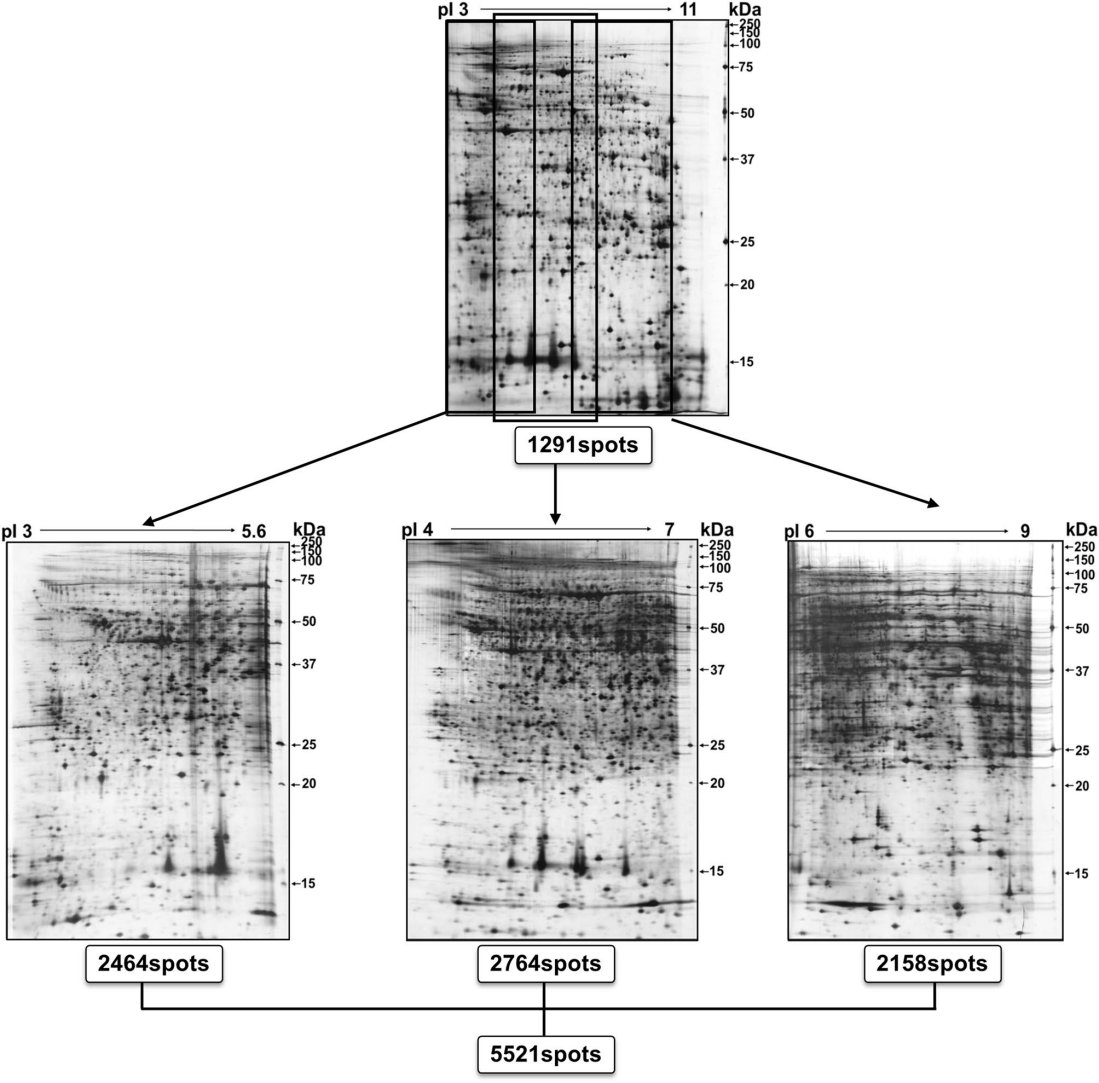
2-DE analysis of total protein in rat kidney tissue. Analysis was performed using four different pI strip ranges (3–11, 3–5.6, 4–7, and 6–9). Proteins were profiled using large gels (35 cm × 45 cm) system and visualized by silver staining.

**Figure 3:**
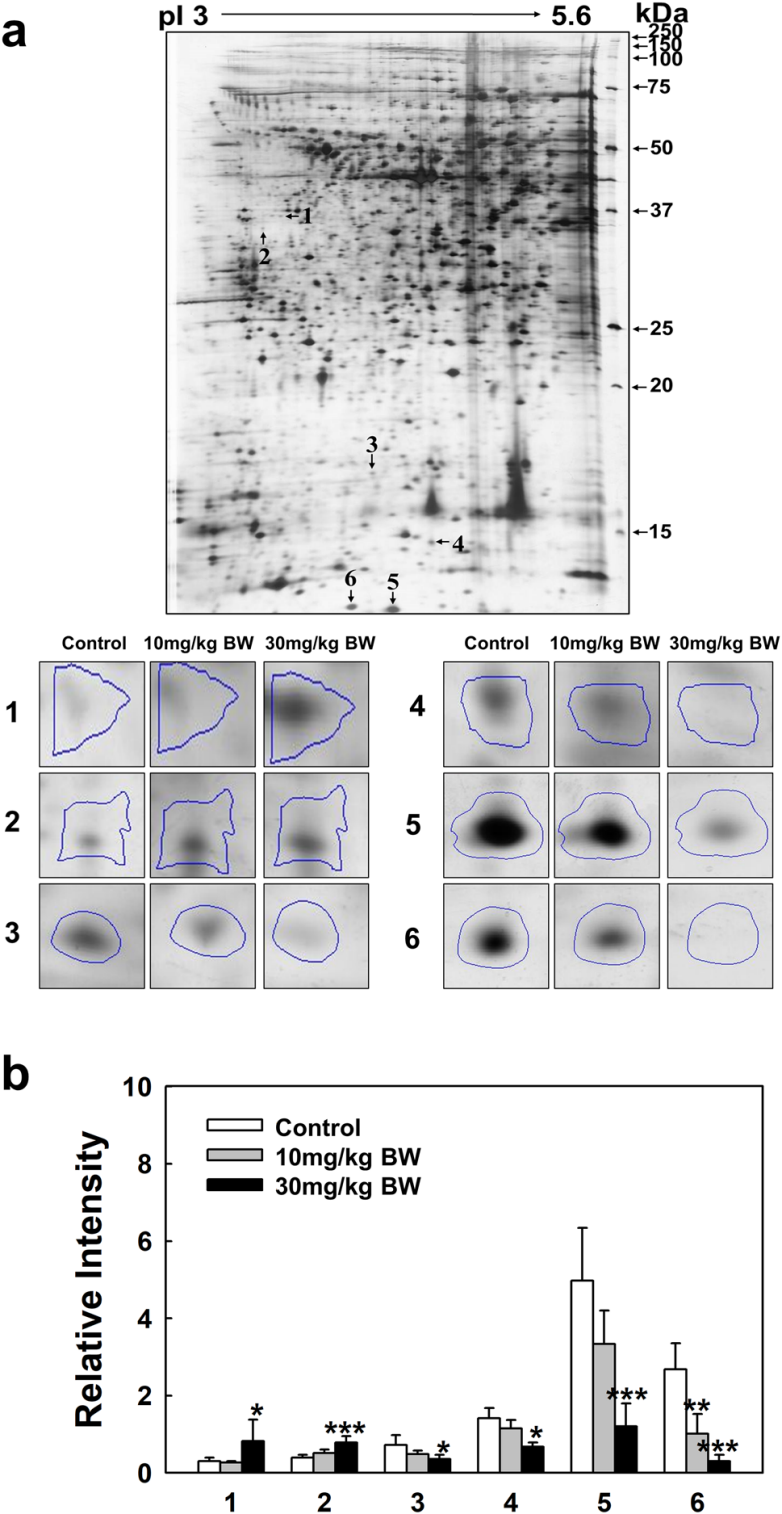
2-DE analysis of kidney tissue proteins obtained using 3–5.6 pI range strips. (**a**) Gel pattern of tissue proteins obtained using 3–5.6 pI strips. Protein spot images were analyzed using the Progenesis Samespot software program (Nonlinear Dynamics, Newcastle upon Tyne, UK). (**b**) Spot volumes were normalized by comparison to the total spot volume. The quantity of each spot is presented as the relative intensity. The images represent the mean ± SD of five separate experiments. *, **, and *** indicate *p*-values of 0.05, 0.01, and 0.001, respectively compared to the control.

**Figure 4:**
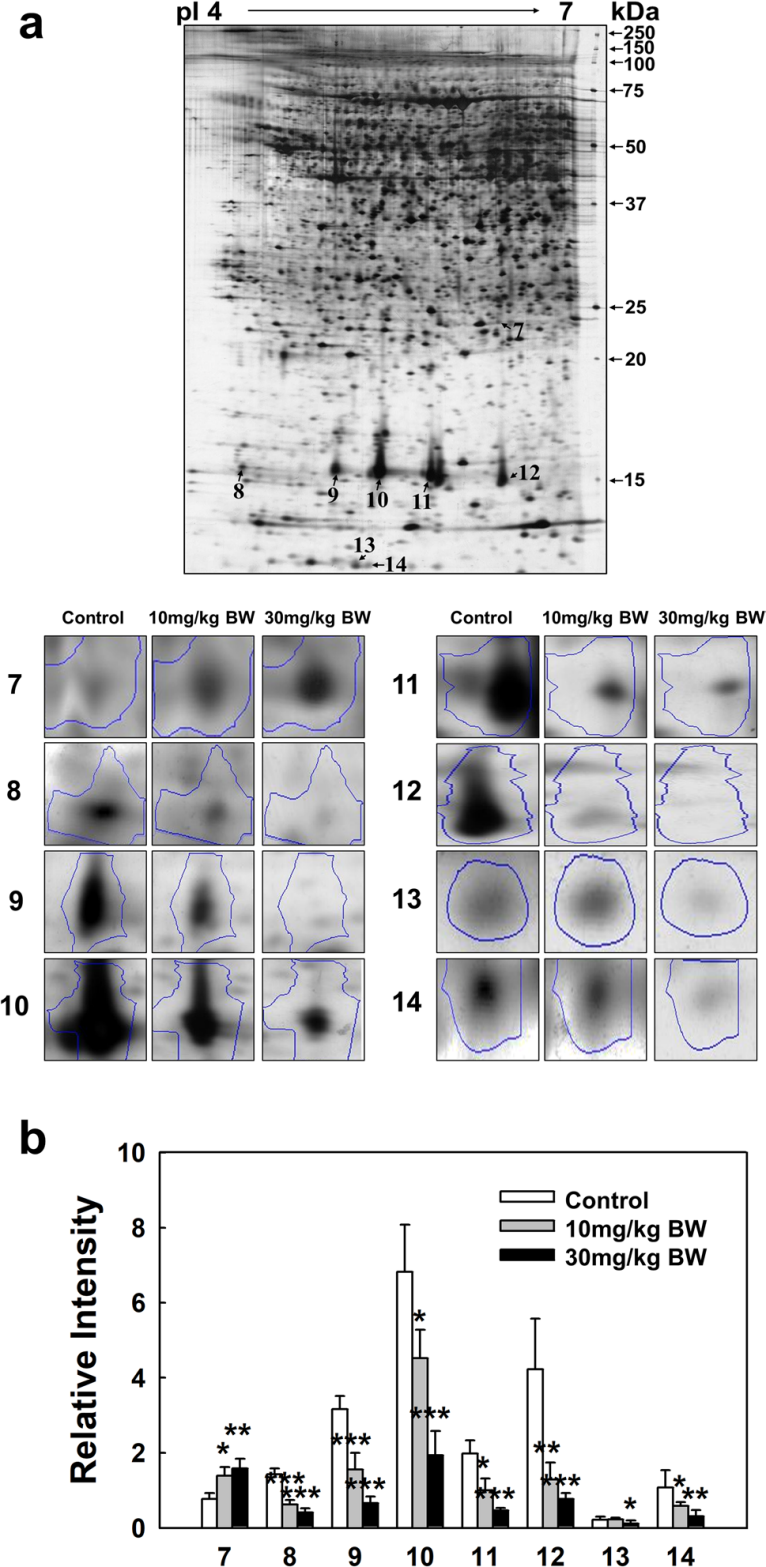
2-DE analysis of kidney tissue proteins obtained using 4–7 pI range strips. (**a**) Gel pattern of tissue proteins obtained using 4–7 pI strips. Protein spot images were analyzed using the Progenesis Samespot software program (Nonlinear Dynamics, Newcastle upon Tyne, UK). (**b**) Spot volumes were normalized by comparison to the total spot volume. The quantity of each spot is presented as the relative intensity. The images represent the mean ± SD of five separate experiments. *, **, and *** indicate *p*-values of 0.05, 0.01, and 0.001, respectively, compared to the control.

**Figure 5:**
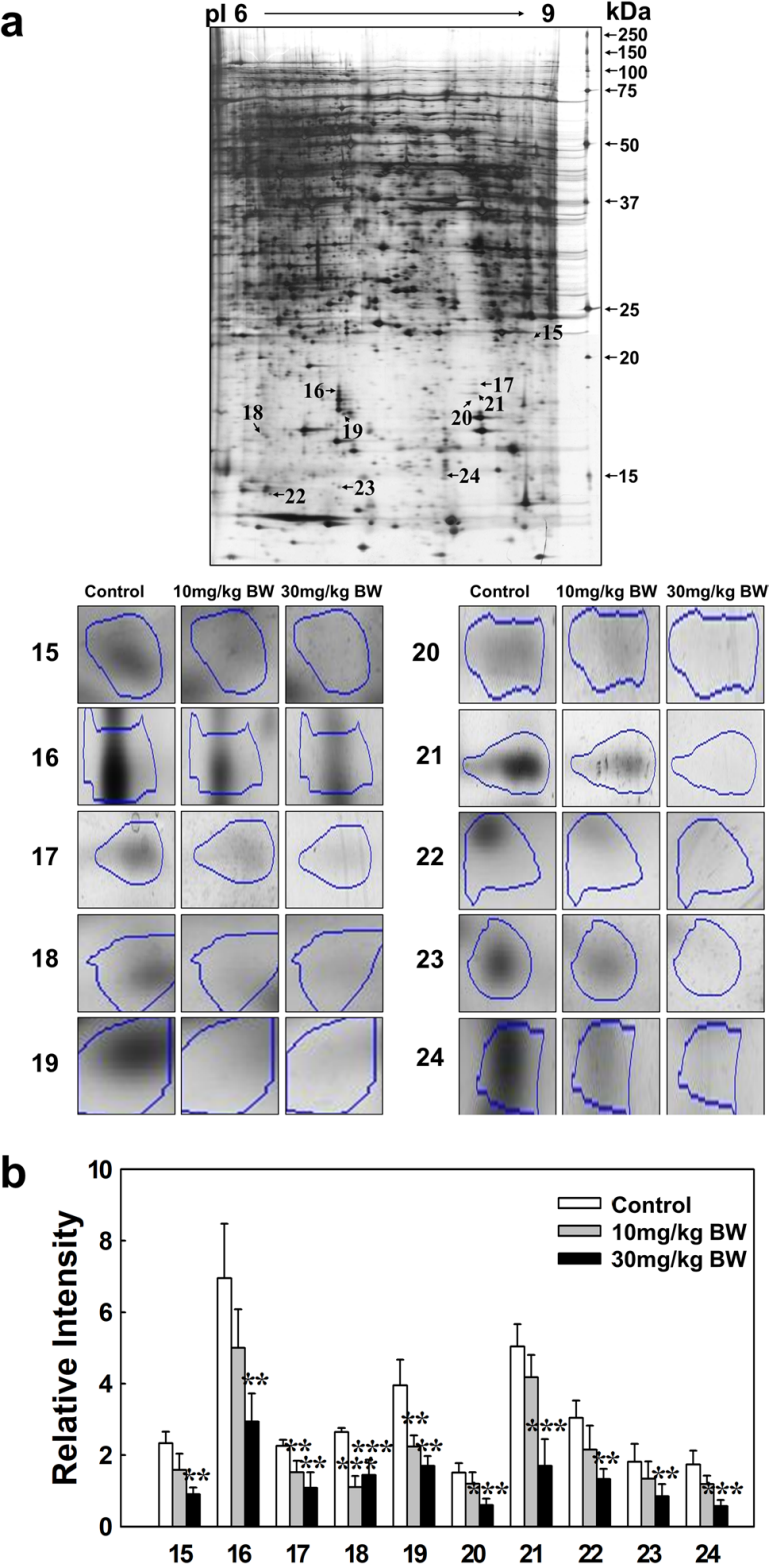
2-DE analysis of kidney tissue proteins obtained using 6–9 pI range strips. (**a**) Gel pattern of tissue proteins obtained using 6–9 pI strips. Protein spot images were analyzed using the Progenesis Samespot software program (Nonlinear Dynamics, Newcastle upon Tyne, UK). (**b**) Spot volumes were normalized by comparison to the total spot volume. The quantity of each spot is presented as the relative intensity. The images represent the mean ± SD of five separate experiments. ** and *** indicate *p*-values of 0.01 and 0.001, respectively, compared to the control.

Proteomic analysis was conducted using four different pI ranges (3–11, 3–5.6, 4–7, and 6–9) and a large size 2-DE system (Fig. 2). The administration of two TAA concentrations (10 mg/kg BW, 30 mg/kg BW) to SD rats for consecutive 28 days was used to identify the biological marker proteins expressed in kidney tissue. Figure 2 shows the 2-DE patterns of proteins expressed in the kidney tissue of rats exposed to TAA using three different ranges of pI strips (3–5.6, 4–7, and 6–9) where 2464, 2764, and 2158 protein spots were present in the gels, respectively (Supplementary image and original gel data). Here, 1420 spots overlapped between the 3–5.6 and 4–7 pI ranges, and 899 spots overlapped between the 4–7 and 6–9 pI ranges (Supplementary Fig. S3). Thus, a total of 5221 proteins spots were resolved (Fig. 2). Of these, 24 expressed proteins were found to be up- and downregulated in the 3–5.6, 4–7, and 6–9 pI ranges (Figs. 3, 4, 5). Specifically, 3 and 21 protein spots were up- and downregulated in a dose-dependent manner, respectively (Figs. 3, 4, 5). Original gels are presented in Supplementary image and original gel data.

### Protein identification and confirmation

**Figure 6:**
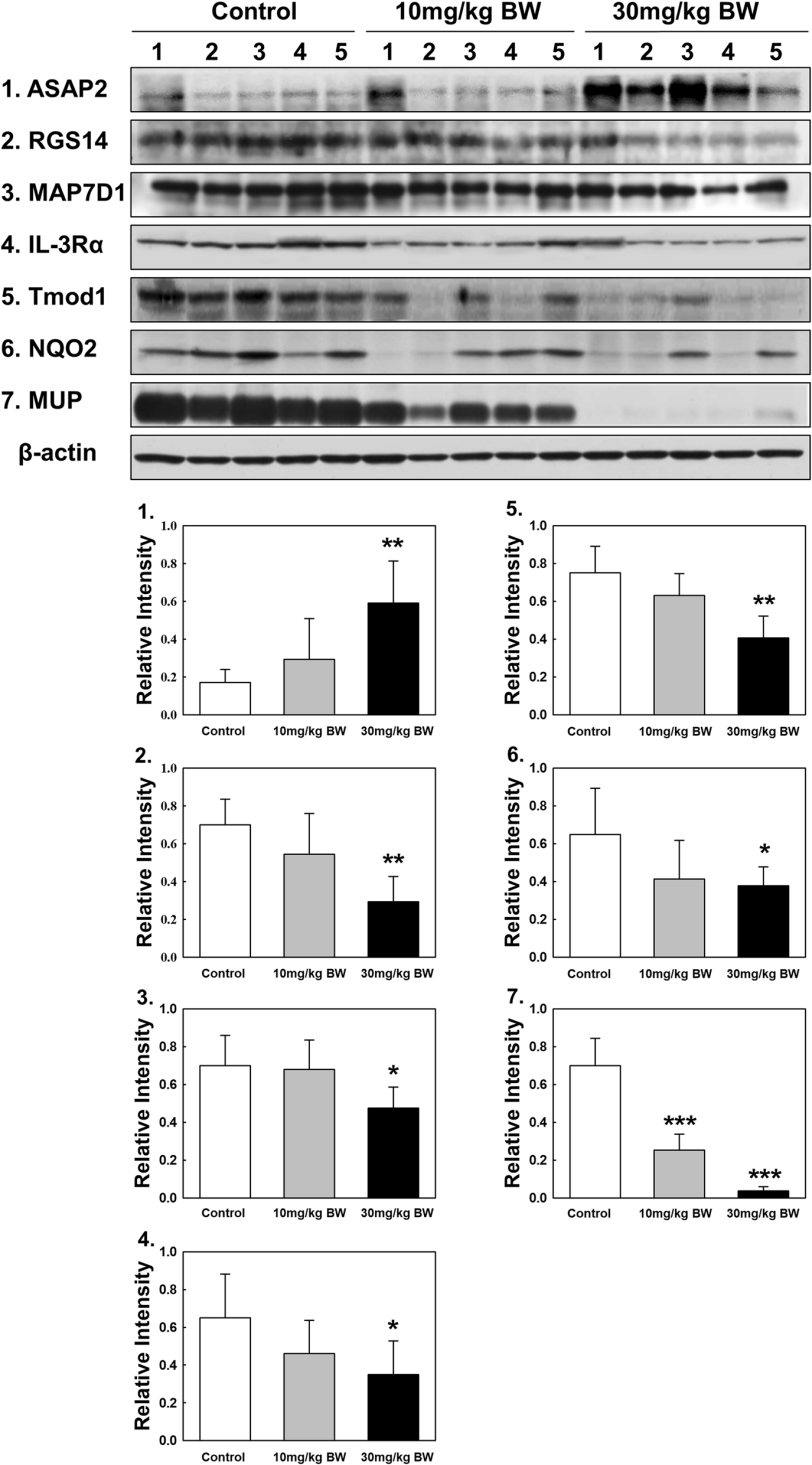
Confirmation of identified proteins by Western blots analysis. (1) ASAP2, (2) RGS14, (3) MAP7D1, (4) IL-3Rα, (5) Tmod1, (6) NQO2, and (7) MUP. Original blots are presented in Supplementary Western blot data. In Western blot analyses, protein samples were subjected to SDS-PAGE with a Western blot kit. Proteins on gels were then transferred onto PVDF membranes. PVDF membranes containing all protein bands were cut prior to hybridization with antibodies. Quantities represented by the gel bands are expressed as intensity relative to β-actin. All relative intensity results are presented as the means ± SD of five experiments. *, **, and *** indicate *p*-values of 0.05, 0.01, and 0.001, respectively compared to the control.

In the 5221 protein spots, 24 were up- or downregulated in kidney tissue in the TAA-administrated group. The protein spots identified by nano-ultra performance LC-ESI-MS^E^ are summarized in Table 3 (Supplementary protein identification data). These proteins were involved in metabolism, the stress response, signal transduction, the immune response, cytoskeleton regulation and organization, protein regulation, cancer, cell growth, other processes (Table 3). Of the 24 proteins differentially expressed in kidney tissue, a total of seven proteins were validated by Western blot analysis. The expression level of Arf-GAP with SH3 domain, ANK repeat and PH domain-containing protein 2 (ASAP2) was significantly upregulated, while most of the other proteins were reduced (Fig. 6). In the case of MUPs, only the expression of the major MUP band was analyzed. MUP was significantly dose-dependently downregulated in the TAA-treated groups compared to the control group (Fig. 6). Original blots are presented in Supplementary Western blot data.

**Table 3:** UPLC-ESI-MS^E^ identification of dose-dependent protein spots in the kidney tissue protein of rats administrated TAA.

Spot no	Accession no	Protein name	Fold change (mg/kg BW)		Matched peptides	Theoretical value (Mr)	Sequence coverage (%)
			10	30			
1	F1LZT6	Arf-GAP with SH3 domain, ANK repeat and PH domain-containing protein 2	+ 0.87	+ 2.69	9	75	7.46
2	G3V6P6	Putative RNA binding protein 3	+ 1.30	+ 2.00	8	13	21.79
3	D4ACM1	Elongator complex protein 3	− 1.49	− 2.03	4	52	8.48
4	P70567	Tropomodulin 1	− 1.23	− 2.10	3	30	11.7
5	P11598	Protein disulfide isomerase A3	− 1.49	− 4.14	16	53	15.84
6	P62749	Hippocalcin like protein 1	− 2.65	− 8.81	7	20	32.12
7	P14942	Glutathione S transferase alpha 4	+ 1.80	+ 2.04	11	20	23.42
8	F1M0B2	Uncharacterized protein Fragment	− 2.29	− 3.49	18	41	11.59
9	P02761	Major urinary protein	− 2.03	− 4.81	24	20	55.24
10	P02761	Major urinary protein	− 1.51	− 3.53	35	20	56.91
11	P02761	Major urinary protein	− 1.98	− 4.27	25	20	61.87
12	Q6AY80	Ribosyldihydronicotinamide dehydrogenase quinone	− 3.27	− 5.51	3	20	17.32
13	P70709	Eosinophil cationic protein (Rnase3)	− 0.96	− 1.99	4	8	25.16
		Eosinophil cationic protein	− 0.96	− 1.99	4	8	25.16
14	F7FIH7	Protein LOC100909412	− 1.85	− 3.50	13	19	38.12
15	F1M6M6	Protein 1700008I05Rik Fragment	− 1.47	− 2.57	2	27	16.59
16	Q5XI73	Rho GDP dissociation inhibitor 1	− 1.39	− 2.36	2	18	12.75
17	Q8VI32	Activated B cell RT1Bl alpha chain	− 1.49	− 2.09	2	13	20.7
18	Q68J51	AC1147 Fragment	− 2.40	− 1.83	3	7	53.68
19	D4AEG9	Homeobox expressed in ES cells 1	− 1.76	− 2.33	3	23	19.46
20	A0JN29	Limb and neural patterns	− 1.26	− 2.52	6	33	13.83
21	O08773	Regulator of G protein signaling 14	− 1.21	− 2.96	8	46	10.66
22	E9PSX4	Interleukin 3 receptor, alpha	− 1.42	− 2.29	5	28	26.42
23	F1LP66	Mitogen activated protein kinase 8	− 1.36	− 2.14	3	37	14.08
24	D4A644	MAP7 domain containing 1	− 1.46	− 3.05	5	78	5.14

### The expression of MUP isoforms in rat kidney exposed to TAA using 2-DE immunoblot assay

**Figure 7:**
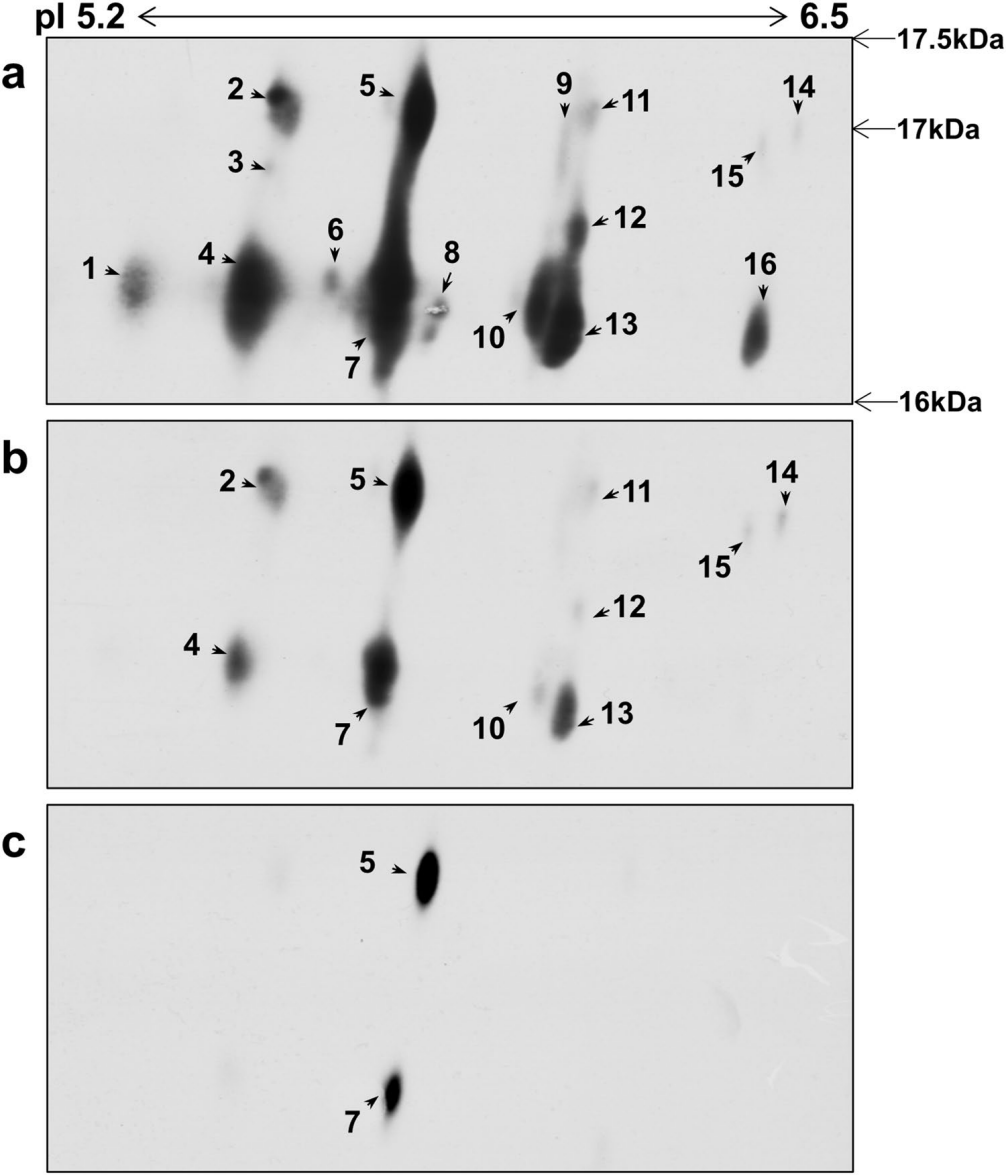
Expression of MUP isoforms in kidneys of rats exposed to TAA using the 2-DE immunoblot Assay. The expression of MUP isoforms was identified by the 2-DE immunoblot assay in the kidneys of rats exposed to TAA. MUP isoforms were separated by 2-DE using pH 4–7 NL (24 cm) strips and 11.5% SDS-PAGE (**a** control group (0.5% carboxymethyl cellulose vehicle only), **b** 10 mg/kg BW TAA, and **c** 30 mg/kg BW TAA). Proteins (pI range from 5.2 to 6.5; MW from 16 to 17.5) were electroblotted onto PVDF membranes (20 × 14 cm). Sixteen isoforms were found to be downregulated with exposure to increasing TAA dosages.

A 2-DE immunoblot assay using pH 4–7 NL (24 cm) strips and 11.5% SDS-PAGE was conducted to determine the expression pattern of the MUP isoforms (Fig. 7). Sixteen isoforms of MUP were found and significantly downregulated with increasing TAA treatment (Fig. 7). Among them, 10 MUP isoforms were found in the kidneys of rats exposed to TAA (10 mg/kg BW) and only two MUP isoforms were found in kidneys of rats exposed to TAA (30 mg/kg BW) (Fig. 7).

### Identification of MUP isoforms in liver, kidneys, and urine in untreated rats by 1-DE Western blot and 2-DE immunoblot assays

**Figure 8:**
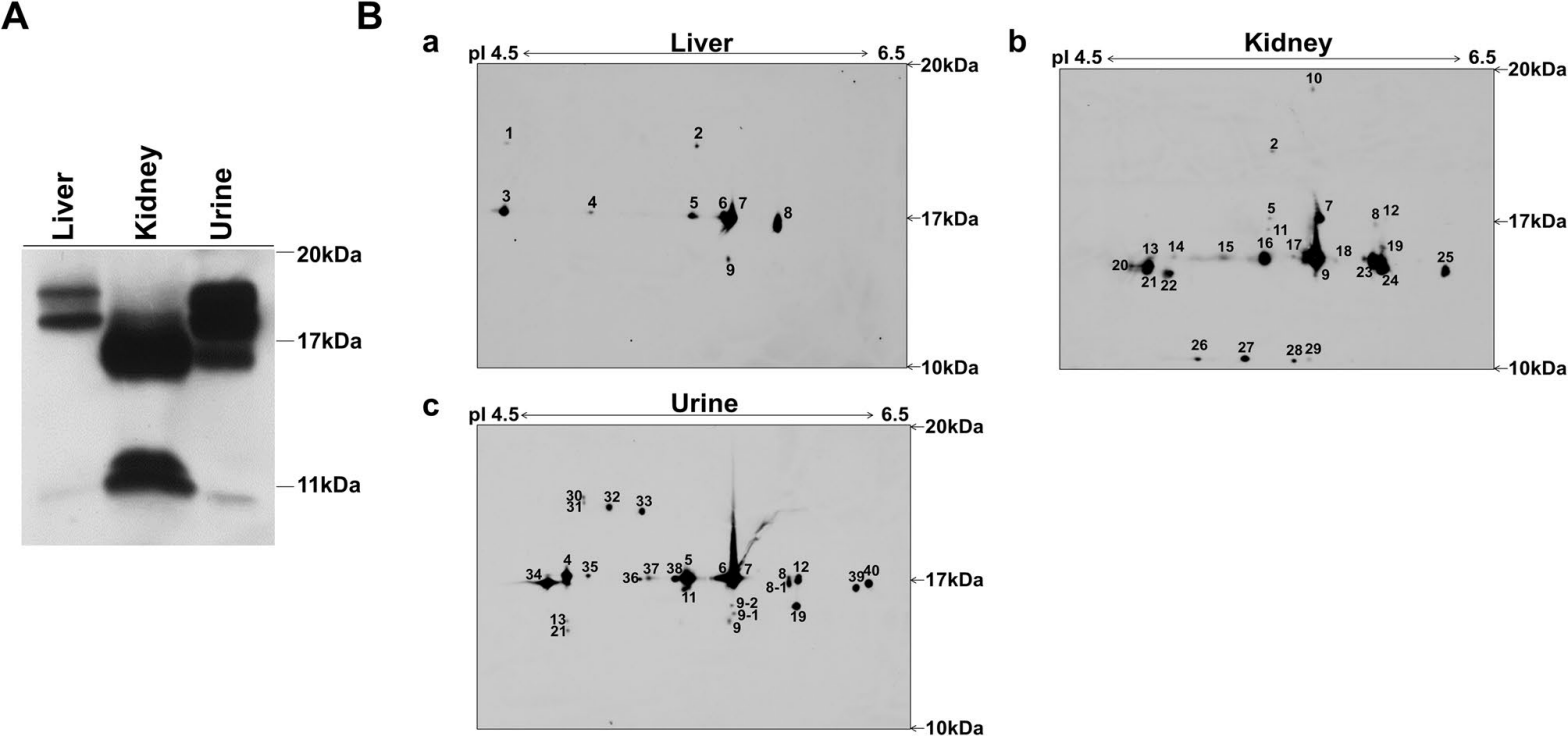
Identification of MUP isoforms in liver, kidneys, and urine in untreated rats by 1-DE Western blot and 2-DE immunoblot assays. (**A**) Comparison of MUP expression in the liver, kidneys, and urine by 1-DE Western blot analysis. Liver (10 μg), kidney (5 μg), and urine (0.0625 μg) proteins obtained from untreated rats were analyzed by 11.5% SDS-PAGE. (**B**) MUP isoforms in the liver, kidneys, and urine by 2-DE immunoblot assay. Liver, kidney, and urine proteins (600 μg, 300 μg, and 3 μg, respectively) obtained from untreated male rats were separated by 2-DE using pH 4–7 NL (24 cm) strips and 11.5% SDS-PAGE. Proteins (pI range from 4.5 to 6.5 pI; MW range from 10 to 20 kDa) were electroblotted onto PVDF membranes (20 × 14 cm). A total of 43 isoforms were found in the liver (**a**), kidneys (**b**), and urine (**c**).

Different amounts of proteins from the liver (10 μg), kidneys (5 μg), and urine (0.0625 μg) were subjected to Western blot assays to identify the 1-DE MUP patterns. Several bands were found in all samples, but the MUP band patterns were different between the liver and kidneys. However, the urine contained the most MUP protein bands (Fig. 8A). In 2-DE immunoblot analysis, liver, kidney, and urine proteins (600 μg, 300 μg, and 3 μg, respectively) obtained from untreated rats were subjected to identify MUP isoforms using pH 4–7 NL (24 cm) strips and 11.5% sodium dodecyl sulfate- polyacrylamide gel electrophoresis (SDS-PAGE). Proteins (pI range 4.5 to 6.5; MW range 15 to 20) were electroblotted onto polyvinylidene fluoride (PVDF) membranes (20 × 14 cm). Several isoforms overlapped in the liver, kidneys, and urine. Two protein spots (number 8 and 9) in the liver were separated into two or three different spots in the urine. A total 43 isoforms were found between the liver, kidneys, and urine (Fig. 8B).

## Discussion

In this study, we determined the body weight and relative organ weight to body weight of rats exposed to TAA to evaluate TAA general toxicity. The body weight decreased, however, the relative organ weights including those of the liver and kidneys were increased. A previous report suggested that a reduction in body weight of TAA-treated rats might be due, in part, to gastrointestinal toxicity and the concomitant loss of the animal’s appetite with subsequent reduction in food intake or the excessive loss of water, salts, and proteins as a result of renal injury, resulting in dehydration and weight loss^21^. In addition, the enlarged livers in the TAA-treated rats indicated hepatic lesions and liver injury associated with the toxicological effects of TAA^21^. In our study, the relative liver and kidney weight to body weight showed similar results of significant increases in rats exposed to the high concentration of TAA.

In a previous study, TAA-treated rats showed leukocytosis, granulocytosis, and thrombocytopenia with decreasing red blood cell (RBC) and MCV and increasing WBCs and PLTs^22^. In our study of the leukocytic parameters, the total WBC counts in rats exposed to TAA were not changed by TAA exposure. However, the numbers of MOs and PLTs were significantly increased in exposed rats compared to the controls. MOs are the largest type of leukocytes and can differentiate into macrophages and conventional dendritic cells. In a previous study, ionized calcium-binding adaptor molecule 1 and galectin-3, which are associated with increasing numbers of CD68 + and CD163 + macrophages, were highly expressed in TAA-induced acute rat liver lesions^23^. Furthermore, the platelet-activating factor levels were significantly increased in rat blood for one month after the administration of TAA in the drinking water (300 mg/L)^24^. In erythrocytic parameters, the MCV and HCT values showed similar results, with significant decreases in rats exposed to the high concentration of TAA.

To evaluate the pathological toxic effects in the kidneys of rats exposed to TAA, we performed histopathological observations of basophilia, casts, cysts, inflammatory cell foci, and interstitial fibrosis in kidney tissue. TAA has shown pathological toxic effects in kidneys, resulting in the deposition of collagen in the renal medulla and fibrin in the tubules^8^, cell death in the terminal portion of the proximal renal tubules^7^, and the renal tissue infiltration of inflammatory cells, degeneration, sclerosis and necrosis of the glomeruli, interstitial fibrosis, and epithelial shedding^11^. However, no significant histopathologic abnormalities were found in the kidneys of rats exposed to TAA in the present study (Fig. 1 and Supplementary Table S1). Based on previous pathological studies, a low dose of 25 mg/kg BW was the highest dose that might lead to mild or no kidney injury, but a high dose of 100 mg/kg BW was the minimum dose of TAA that could result in organ injury^7,8,11,25^.

Therefore, further biochemical studies were performed to evaluate the toxic effects of TAA in the kidneys of rats exposed to TAA. A panel of novel urinary kidney biomarkers was recently approved for the improved detection of acute nephrotoxicity by the U.S. Food and Drug Administration, the European Medicines Agency, and the Pharmaceuticals and Medical Devices Agency (Japan)^26^. Among them, Fuchs et al. proposed that the most promising biomarkers were NGAL, Kim-1, osteopontin, clusterin, RPA-1, and GSTYb1, detected by multiplexing technologies^27^. In this study, four protein biomarkers including Kim-1, NGAL, osteopontin, and clusterin, which are biomarkers of injury to the proximal and distal tubules^26^, were applied to evaluate the nephrotoxicity of TAA using Western blot assays. The expression levels of Kim-1 and NGAL were significantly increased in the kidneys and urine of rats exposed to the high concentration of TAA, resulting in significant nephrotoxicity.

In proteomic analysis, 5221 proteins in the kidneys of rats exposed to TAA were analyzed to determine the protein changes. Twenty-four proteins were significantly up- and downregulated. Of these, the expression of seven proteins including ASAP2, regulator of G protein signaling 14 (RGS14), MAP7 domain containing 1 (MAP7D1), interleukin 3 receptor, alpha (IL-3Rα), Tropomodulin 1 (Tmod1), N-ribosyldihydronicotinamide dehydrogenase quinone (NQO2), and MUP were validated by Western blot assays.

ASAP2 encodes for a 1006-amino acid multi-domain protein and is involved in the regulation of vesicular transport, cellular migration, and autophagy^28,29^. ASAP2 was also reported to promote tumor growth by facilitating cell cycle progression by phosphorylating the epidermal growth factor receptor^30^. In this study, the expression levels of ASAP2 showed a threefold increase in the kidney tissue of rats exposed to the high concentration of TAA compared to the control group. TAA has been known as a carcinogen, which might be involved in the activation of cancer cell signaling.

Regulators of G protein signaling (RGS) are intracellular signaling regulators^31–33^. Among the RGS proteins, RGS14 is a highly unusual RGS protein with a multidomain structure that allows it to interact with binding partners from multiple signaling pathways^34^. It has been reported that RGS14, an upstream regulator of the AC-PKA and Raf⁄MEK⁄ERK signaling pathways, functions globally in stress resistance and the longevity of several species, beyond its specific cellular functions. When RGS14 expression was reduced in rat fibroblast cells, the resistance to oxidative stress increased with higher MnSOD expression^35,36^. In this study, the expression levels of RGS 14 were significantly decreased in the kidney tissue of rats exposed to the high concentration of TAA compared to the control group. A decrease in RGS14 expression by TAA may reduce the resistance to oxidative stress and renal cellular longevity.

Microtubule-associated proteins (MAPs) are a family of proteins that bind to and stabilize microtubules^37,38^. Four MAP7 paralogs, MAP7, MAP7D1, MAP7D2, and MAP7D3, are encoded in the mammalian genome, and phylogenetic analysis suggested that MAP7D1 was the most conserved MAP7^39,40^. MAP7 may enhance the membrane expression of transient receptor potential vanilloid 4 (TRPV4) and possibly, link cytoskeletal microfilaments. In renal epithelial cells, TRPV4 channel activation results mainly from hypotonic cell swelling, which suggests that TRPV4 acts as an osmosensor and could play a critical role in renal ischemic/reperfusion injury^41,42^ and in autosomal recessive polycystic kidney disease^43^. TRPV4 dysfunction promotes renal cystogenesis in autosomal recessive polycystic kidney disease^43^. In this study, the expression levels of MAP7D1 were significantly decreased in the kidney tissue of rats exposed to the high concentration of TAA compared to the control group. TA might be involved in kidney damage, causing TRPV4 dysfunction by decreasing the expression of MAP7D1.

IL-3Rα, which is also called CD123, is a cell surface protein that is widely expressed across the various subtypes of acute leukemia^44–46^. Many studies have been published in the immunology, signaling pathway, hematological malignancy, and immunotherapy fields^44–49^. However, few studies have evaluated the renal toxicology of IL-3Rα. In the present study, IL-3Rα was significantly downregulated in the kidney tissue of rats exposed to the high concentration of TAA. The lower expression of IL-3Rα by TAA may reduce the heterodimer formation of IL-3 with other receptors, which might inhibit physiological functions including cell proliferation and survival.

Tmods are unique actin-binding proteins that cap the slow-growing ends of actin filaments, a major component of the cytoskeleton^50–52^. Recently, Wang et al. found that Tmod1 was specifically expressed in distal tubules and the collecting ducts of the kidney that regulate water homeostasis. Furthermore, Tmod1 was found to be closely related to metabolic processes, protein phosphorylation, and multiple signaling pathways by proteomic and bioinformatic analyses^53^. These results indicate the critical role of Tmod1 in renal function and provide new molecular mechanisms for the regulation of water balance^53^. In the present study, Tmod1 was significantly downregulated in the kidneys of rats exposed to the high concentration of TAA. The higher expression of the two kidney injury biomarkers Kim-1 and NGAL showed that TAA caused injury to the kidney proximal and distal tubules. Therefore, the significant downregulation of Tmod1 showed that TAA may play a critical role in kidney function.

NQO2 is a cytosolic enzyme. The initial Northern blot findings in human specimens indicated that the highest expression was in skeletal muscle followed by kidneys, liver, lungs, and heart^54,55^. In previous studies using an NQO2 knockdown cell line and NQO2 siRNA, the inhibition of NQO2 activity induced the upregulation of antioxidant enzymes, which resulted in increased cellular resistance to oxidants and protected cellular components from oxidation-related damage^56–58^. Furthermore, high concentrations of resveratrol, which is a natural antioxidant agent, induced the downregulation of NQO2 expression, which inhibited the angiotensin II-induced generation of reactive species oxygens in rat vascular smooth muscle cells^57,58^. An immunohistochemical toxicity study showed that TAA significantly reduced the expression level of NQO1 in rat renal tissues^59^. In the present study, NQO2 was significantly downregulated in the kidneys of rats exposed to the high concentration of TAA. The downregulation of NQO2 by TAA exposure might protect renal cellular components against oxidative damage.

MUPs are low-molecular-weight (approximately 19 kDa) members of the large lipocalin family^60–63^. They are synthesized in the liver as precursors and, after excision of the signal peptide and formation of disulfide bonds, the small proteins are secreted into the bloodstream to be finally excreted into the urine^60–63^. MUPs are also expressed in other tissues, including the salivary, lachrymal, meibomian, mammary preputial, and perianal glands, nasal tissue, and respiratory epithelia^60–63^.

In the present study, MUPs were significantly downregulated in the kidneys of rats exposed to the high concentration of TAA. The downregulation of MUPs in kidney tissue could be caused by TAA hepatotoxicity because MUP is mostly synthesized in the liver and TAA induced a significant downregulation of MUPs in the liver of rats exposed to TAA (Supplementary Fig. S4). On the other hand, MUP down-regulation might be attenuated by the renal injury of kidney proximal and distal tubules, resulting in the inhibition of MUP resorption. It has been reported that MUPs were reabsorbed by kidney renal tubule cells by the general mechanism for low-molecular-weight proteins^64^. In this study, we found three MUP isoforms, which were located in the 5.0 pI to 5.75 pI range, and they were all significantly downregulated. However, in a previous proteomic study, which showed protein expression level changes in rat livers exposed to TAA, three major urinary proteins were present as isoforms with pI ranges of 5.19, 5.29, and 5.43. Among them, two MUPs were significantly downregulated, but one major urinary protein remained unchanged^65^. Therefore, we evaluated the changes in MUP isoforms in the kidney tissue of rats exposed to TAA using the 2-DE immunoblot assay with a large polyacrylamide gel (size 35 × 45 cm) with a pI range from 5.2 to 6.5 and a molecular weight range from 16 kDa to 17.5 kDa. Sixteen MUP isoforms were found across the pI ranges and they were all significantly downregulated by exposure to increasing concentrations of TAA (Fig. 7).

In previous studies, IEF of male mouse urine resolved up to 15 MUP isoforms in a pI range from 4.6 to 5.3^60,61^. To identify all MUP isoforms in kidney tissues, the 2-DE immunoblot assay was applied with extended pI ranges from 4.5 to 6.5 and molecular weights from 10 to 20 kDa (Fig. 8B). Furthermore, MUP isoform patterns in the liver, kidneys, and urine were compared. In this study, different amounts of protein samples from the liver, kidneys, and urine obtained from untreated male rats were used for the fine separation of protein bands and spots in 1-DE Western blots and 2-DE immunoblot assays (Fig. 8). In the 1-DE Western blot assay, liver, kidney, and urine showed different protein bands (Fig. 8A). One protein band in the liver was barely visible in the kidneys and the other two protein bands showed more intensity in the kidneys than in the liver. However, the urine showed all bands, although the low-molecular-weight protein bands were only slightly seen. It has been reported that 60% of MUPs, which are synthesized in the liver, were reabsorbed in the proximal tubule and degraded in the lysosomal compartment in the kidney, resulting in the generation of proteins truncated at ∼15.5 kDa, which accumulated in the cytosol^66^. In contrast, a different pattern of protein bands between the liver and kidneys might be derived from MUPs that were synthesized from other tissues including the salivary, lachrymal, meibomian, mammary preputial and perianal glands, nasal tissue, and respiratory epithelia^60–63^. The 2-DE immunoblot assay showed variable patterns of MUP isoforms in the liver, kidneys, and urine, although the major MUP isoform spots were found in all samples (Fig. 8B). To obtain the fine separation of each MUP isoform spot, different amounts of protein samples of the liver, kidneys, and urine were analyzed. This methodological approach could reveal a limitation in comparing the MUP isoform patterns in the liver, kidneys, and urine using the 2-DE immunoblot assay because the small-sized MUP isoforms were not detected in the gel. A total of 43 different MUP isoforms were found in the liver, kidneys, and urine. Previous studies have been performed to evaluate MUP profiles in different kinds of rodents and investigate MUP profile dynamics^62,63,67–71^. The present study also provides additional information for MUP profiles in the synthesis, circulatory, and secretory biological systems of laboratory male rats.

In conclusion, TAA is a carcinogen and a hepatotoxicant and is known as a nephrotoxicant, which induced structural kidney damage including severe tubular epithelial cell death associated with inflammatory cell infiltration, sclerosis and necrosis of the glomeruli, and fibrosis^9,11,13–15^. In this study, we determined the hematological, pathological, and biochemical toxicity of TAA and evaluated the proteomic changes in kidney tissue under different conditions after exposure to TAA. In the hematological study, total WBC counts were not changed by TAA exposure. However, the numbers of MOs and PLTs were significantly increased, and the MCV and HCT values were decreased significantly in rats exposed to 30 mg/kg BW TAA. No significant histopathologic abnormalities were found in the kidneys of rats exposed to TAA. Nonetheless, the expression levels of Kim-1 and NGAL, which are kidney injury biomarkers, showed significant increases in the kidney tissue of rats exposed to TAA, indicating nephrotoxicity. In proteomic analysis, seven proteins including ASAP2, RGS14, MAP7Dl, IL-3Rα, Tmod1, NQO2, and MUP were found to be up- and down-regulated and a total of 43 MUP isoforms were found in the liver, kidneys, and urine.

## Materials and methods

### Chemicals

TAA, formaldehyde, urea, CHAPS, DTT, iodoacetamide, methanol, ethanol, and sodium thiosulfate were purchased from Sigma-Aldrich (Darmstadt, Germany). Bis-acrylamide solution and protease inhibitor cocktail were purchased from Bio-Rad (Hercules, CA, USA). High-purity glacial acetic was purchased from J.T Baker (Loughborough, England). All other chemicals used in this study were the highest grade commercially available.

### Animals

Specific-pathogen-free male, Sprague–Dawley rats aged 6–7 weeks (200–220 g BW were obtained from Orient Bio (Seongnam, Korea) and acclimated for one week. The rats were provided with a commercial pellet diet and tap water ad libitum. TAA was orally administered to the animals daily for 28 consecutive days. A total of 15 rats (5 rats per group) were divided into three groups: 1) control group (0.5% carboxymethyl cellulose as vehicle-only), 2) 10 mg/kg BW TAA, and 3) 30 mg/kg BW TAA. All animals were then housed individually in metabolic cages equipped with urine and feces separators. The rats were fasted overnight and euthanized under isoflurane anesthesia 24 h after the last dosing. Rat liver and kidney tissue and urine samples were collected and frozen in liquid nitrogen for further analysis.

### Ethics statement

All animal experiments were approved by the Institutional Animal Care and Use Committee of Korea Institute of Toxicology (approval #1304-0101) and conducted in accordance with the relevant animal guidelines. The study was carried out in compliance with the ARRIVE guidelines.

### Histopathological analysis

Kidney tissues were fixed in 10% formalin and then embedded in paraffin. Tissue slides of 5-μm-thick sections were stained using hematoxylin and eosin and then observed by light microscopy. Histopathologic changes were scored based on the pathologist's impression. The severity scoring system used in all categories consisted of a scale of 0 to 5, where 0 was not remarkable, 1 was very slight, 2 was slight, 3 was moderate, 4 was marked, and 5 was the highest.

### Hematological analysis

Under isoflurane anesthesia, blood samples were drawn from the ventral aorta using a syringe. The blood samples were collected into EDTA tubes and analyzed within 15 min using an automatic hematology analyzer (Drew Scientific, USA). The following hematological parameters were analyzed: WBC count, PLT count, lymphocyte (LY) count, MO count, eosinophil (EO) count, basophil (BA) count, RBC count, HCT value, MCV, MCH, and mean corpuscular hemoglobin concentration (MCHC).

### Sample preparation and 2-DE large gel PAGE

Sample preparation and 2-DE large gel PAGE analysis were performed as described previously with minor modifications^72^. Kidney and liver tissues finely ground under liquid nitrogen were homogenized by sonication in lysis buffer containing protease inhibitors and the insoluble cellular debris was removed by centrifugation. Protein samples aliquots were prepared as described in the 2D Clean-up Kit (GE Healthcare Life Science, USA), and the pellet of the final step was solubilized in sample buffer containing 7 M urea, 2 M thiourea, 40 mM Tris (0.5 M, pH 8.5), 4% CHAPS, 65 mM DTT, and 1% IPG buffer. Protein concentrations were measured using the Bradford assay (Bio-Rad, USA). Urine was centrifuged at 2500×*g* for 30 min at 4 °C. The supernatant was collected and concentrated using a Microcon 3,000 molecular weight cutoff filter (Millipore, Bedford, MA, USA) following three washes with 300 μl of 50 mM Tris buffer. In the first dimension of the 2-DE, the proteins were separated according to their isoelectric points. The protein sample (250–300 µg) was mixed with a rehydration buffer (GE Healthcare Life Science) to a total volume of 150 µL per sample. Isoelectric focusing (IEF) was carried out with commercially available immobilized pH gradients (pH 3–11 nonlinear, 3–5.6 nonlinear, 4–7 linear, 6–9 linear, 24 cm), using the IPG Phor (Amersham Biotech, Amersham, UK) apparatus. After IEF, the IPG gel strips were equilibrated twice for 15 min under gentle shaking at room temperature, first in a solution (equilibration buffer: 50 mM Tris–HCl, pH 8.8, 6 M urea, 30% glycerol, 1% w/v SDS containing 1% dithiothreitol (DTT), then in an equilibration buffer containing 2.5% iodoacetamide. In the second dimension, SDS-PAGE, the proteins were resolved on 12.5% polyacrylamide gels (size 35 × 45 cm) solely on the basis of their molecular masses using a large-gel separation system runner. The IPG strips were embedded in 0.5% w/v melted agarose prior to running on the SDS-PAGE slabs. The agarose contained 0.001% w/v BPB as a tracking dye. The running conditions were 1 w/gel for 30 min and 20 w/gel for 14–16 h until the BPB reached the end of the gel.

### Visualization and image analysis

Visualization and image analysis were performed as described previously^72^. After separation on SDS-PAGE gels, the proteins were visualized by silver staining according to the manufacturer’s instructions (GE Healthcare). The silver-stained gels were scanned using a 3600 × 4900 dpi instrument (Epson Expression 10000XL, Epson, Japan) and the image files were transformed into TIFF format with linear grayscale values. Computer analysis of the 2DE image was carried out using Progenesis Samespot image analysis software (Nonlinear Dynamics, Newcastle upon Tyne, UK) according to the manufacturer’s protocol. Intensity levels were normalized between gels as a proportion of the total protein intensity detected on the entire gel.

### Identification of proteins by UPLC-ESI-MS^E^

Protein identification and data analysis were performed as described previously^72^. Briefly, separation was carried out using a nano-ACQUITY Ultra Performance LC Chromatography™ System (Waters Corporation, Milford, MA, USA) with a nano-Acquity UPLC BEH130 C18 RP column and an enrichment Symmetry C18 RP column. Five microliters of tryptic-digested peptides (50 mM ammonium bicarbonate, 5 mM calcium chloride, and 12.5 ng/mL trypsin in digestion buffer) in mobile phase A (water with 0.1% formic acid) were loaded onto the column for each experiment and 5–45% mobile phase B (acetonitrile with 0.1% formic acid) was run over 55 min with a step gradient (flow rate of 280 nL/min), followed by an increase to 90% B in 10 min. Tandem mass spectroscopy (MS) of [Glu1]-fibrinopeptide (400 fmol/μL) was used to calibrate the time-of-flight analyzer in the range of 50–1990 m/z. [Glu1]-fibrinopeptide (785.8426 m/z) was used for lock mass correction. During data acquisition, the collision energy of the low-energy MS mode and elevated-energy mode was set to 4 eV and the 15–40 eV energy ramping mode, respectively. One cycle of MS and MS^E^ was performed every 3.2 s. In each cycle, MS spectra were acquired for 1.5 s with a 0.1 s interscan delay (50–1990 m/z), and ions exceeding 50 counts were selected for MS^E^ fragmentation in the collision cell (50–1990 m/z). To generate ion spectra for subsequent database searching, ProteinLynx Global Server version 2.4 was used to process and search the liquid chromatography (LC)-MS^E^ raw data files. Proteins were identified by searching the Homo sapiens protein database on the UniProt website including 70,718 entries. For the ProteinLynx Global Server search, low or high collision spectra were processed with a hierarchical approach, which required detection of at least three fragment ion matches per peptide, seven fragment ion matches per protein, and two peptide matches per protein with a maximum 4% false positive rate. Cysteine carbamidomethylation (+ 57 Da) and methionine oxidation (+ 16 Da) were chosen as the fixed and variable modifications, respectively.

### Western blot assay

Kidney and liver tissue samples were prepared in lysis buffer containing protease inhibitors. Urine sample was prepared using a 3 kDa cut-off membrane. Accurate determination of protein concentration using Bradford assays by manufacture’s instruction (Bio-rad, USA). The protein sample (5-30ug) is diluted with 2X sample loading buffer and denatured by heating for 5 min between 70 and 100 ℃. The gel- separated proteins (5–15%) are transferred and immobilized to a polyvinylidene fluoride (PVDF) membrane. The membranes were blocked (5% skimmilk in PBS-T) for 1 h and incubated with analyte-specific primary antibody for 1 h at room temperature; ASAP2 (Santa Cruz,1:1000), RGS14 (Santa Cruz,1:500), MAP7D1 (Santa Cruz,1:1000), IL-3Rα (Santa Cruz,1:1000), Tmod1 (Santa Cruz,1:500), MUP (Santa Cruz,1:1000), NQO2 (Santa Cruz,1:1000), Kim-1 (R&D systems, 1:1000), NGAL (R&D systems, 1:1000), β-actin (Santa Cruz,1:2000). In this study, four kinds of antibodies including two monoclonal and two polyclonal antibodies were used to evaluate the expression of MUP. Among them, one monoclonal antibody was selected for 1-DE Western blot and 2-DE immunoblot analyses (Supplementary Fig. S5). After washing the membranes with PBS-T three times for 5 min each, they were incubated with HRP-conjugated secondary antibodies [anti-rabbit IgG, anti-goat IgG or anti-mouse IgG (1:2000, Santa Cruz, CA, USA)] for 1 h with rocking and then washed with PBS-T 3times for 20 min. Incubate the membranes in Pierce™ ECL Western Blotting Substrate (Themo fisher, USA) for 5 min with rocking. Bands were visualized by film and scanned using a 3600 × 4900 dpi instrument (Epson Expression 10000XL, Epson, Japan). The digitalized images were analyzed using Image J program.

### Two-dimensional immunoblot assay

For two-dimensional immunoblot analysis of MUPs, rat liver, kidney, and urine proteins were separated by 2-DE using pH 4–7 NL strips and by 11.5% large-size SDS-PAGE. To evaluate the changes in the MUP isoforms in the kidneys of rats exposed to TAA, protein samples obtained from 5 rats (a total of 15 rats from 3 groups) were mixed and 300 ug of protein was used for 2-DE immunoblot analysis. The 2-DE gel, which was located in the pI area from 5.2 to 6 and molecular weight range from 16 kDa to 17.5 kDa, was removed and then electroblotted onto PVDF membranes (20 × 14 cm). To compare the MUP isoforms in liver, kidney and urine, protein samples from 10 untreated male rats (treated only with the vehicle (0.5% carboxymethyl cellulose)) were mixed and used for 2-DE immunoblot analysis. The 2-DE gel, which was located in the pI area from 4.5 to 6.5 and molecular weight range from 10 to 22 kDa was removed and then electroblotted onto PVDF membranes (20 × 14 cm). (Supplementary Fig. S6).

### Statistical analysis

Statistical analysis was performed with IBM SPSS statistics version 22.0 software (IBM, Inc., Chicago, IL, USA). All data are expressed as the mean ± SD. Between-group differences were analyzed by the non-parametric Mann–Whitney U test. Significance is indicated in the figures and tables as *p < 0.05, **p < 0.01, and ***p < 0.001.

## Supplementary Information

Supplementary Information.
