# Supplementary material for: Nephrotoxicity evaluation and proteomic analysis in kidneys of rats exposed to thioacetamide
Source: Sci Rep. 2022 Apr 27;12:6837. doi: 10.1038/s41598-022-11011-3 (PMC9046159; doi:10.1038/s41598-022-11011-3)
Supplement: Supplementary file 1 — Supplementary Information. [file 41598_2022_11011_MOESM1_ESM.pdf]

Nephrotoxicity evaluation and proteomic analysis in kidneys of rats exposed to thioacetamide

Ji-youn Lim[a,1], Woon-Won Jung[b], Woojin Kim[c], Kyoung-Sik Moon[c,]**, Donggeun Sul[a,]*

[a]Graduate School of Medicine, Korea University, Seoul 136-705, Republic of Korea

[b]Department of Biomedical Laboratory Science, College of Health Science, Cheongju University, Cheongju 28503, Republic of Korea

[c]Department of Advanced Toxicology, Korea Institute of Toxicology, Daejeon 34114, Republic of Korea

[1]Present Address: New Drug Development Center, Daegu-Gyeongbuk Medical Innovation Foundation, Daegu 41061, Republic of Korea

*Corresponding author: Donggeun Sul, Ph.D, Graduate School of Medicine, Korea University, 73 Inchon-ro, Sungbuk-Ku, Seoul 136-705, Republic of Korea, E-mail address: dsul@korea.ac.kr.

**Additional corresponding author: Kyoung-Sik Moon, Ph.D, Department of Advanced Toxicology, Korea Institute of Toxicology, 141 Gajeong-ro, Yuseong-Ku, Daejeon 34114, Republic of Korea, E-mail: ksmoon@kitox.re.kr

Journal: Scientific Reports

Supplementary Information

Supplementary Table S1 Histopathological observation of the kidney of rats exposed to TAA

Histopathological change	Thioacetamide (mg/kg BW)		
	0	10	30
Basophilia	1 (1/5)*	1 (2/5)*	1 (3/5)*
Cast	0	0	0
Cyst	0	0	0
Inflammatory cell foci	0	1 (1/5)*	0
Interstitial fibrosis	0	0	0

Histopathological severity (0 = not remarkable, 1 = very slight, 2 = slight, 3 = moderate, 4 = marked, and 5 = highest). *number of rats that showed the histopathological severity out of 5 rats.

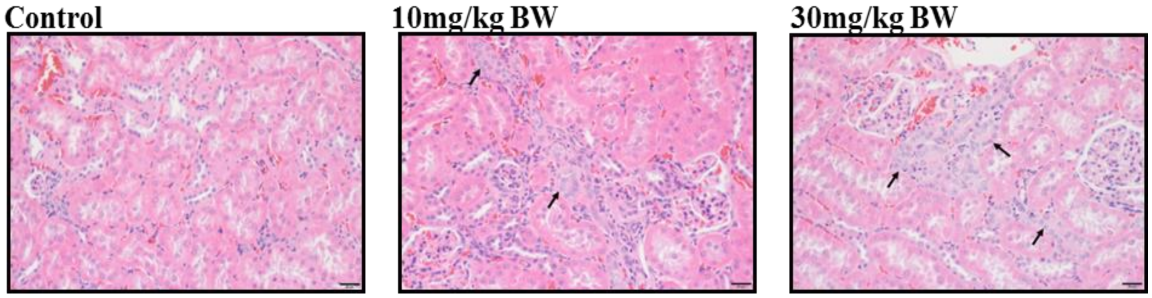

Fig. S1 Histopathological observations in kidneys of rats exposed to thioacetamide. Kidney tissues were fixed in 10% formalin and then embedded in paraffin. Tissue slides of 5-μm-thick sections were stained with hematoxylin and eosin and then observed by light microscopy (scale bar = 20 μm; magnification, x200). Control group (0.5% carboxymethyl cellulose as vehicle only), 10 mg/kg BW TAA, 30 mg/kg BW TAA. Arrow: basophilia.

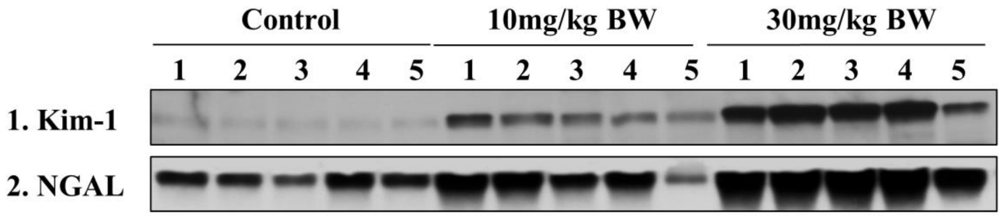

Fig. S2 Western blot analysis of Kim-1 and NGAL proteins in the urine of rats exposed to TAA. Control group (0.5 % carboxymethyl cellulose vehicle only), 10 mg/kg BW TAA, and 30 mg/kg BW TAA.

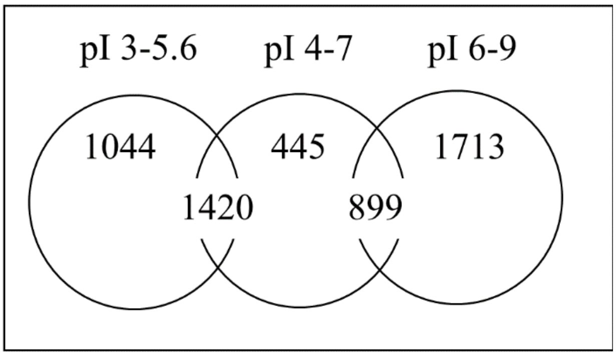

Fig. S3 Overlapping protein spots in the gels with pI strips. A total of1420 protein spots overlapped between the 3-5.6 and 4-7 pI ranges and 899 spots overlapped between the 4-7 and 6-9 pI ranges. A total of 5221 proteins spots were resolved.

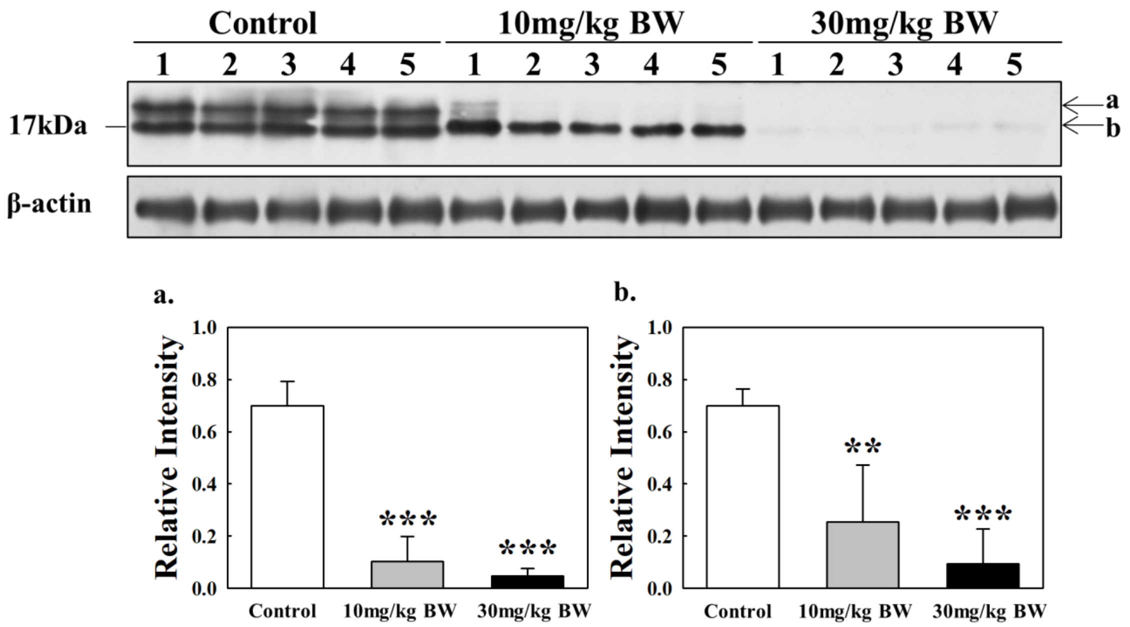

Fig. S4 Western blot analysis of MUPs in the liver of rats exposed to TAA. The quantities represented by the gel bands are expressed as intensity relative to β-actin. All relative intensity results are presented as means ± SD of five experiments. ** and *** indicate *p*-values of 0.01 and 0.001, respectively, compared to the control.

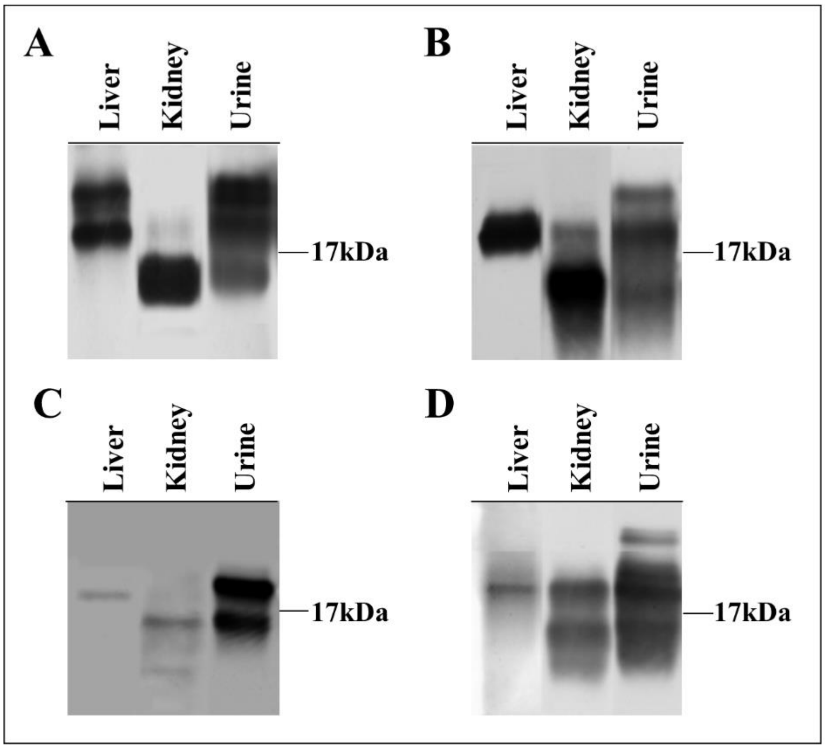

Fig. S5 Western blot analysis of MUPs in the liver, kidneys, and urine using four antibodies. Four mono and polyclonal antibodies were used to determine the 1-DE pattern of MUPs in the liver, kidneys, and urine. **A** Monoclonal antibody from Santa Cruz, **B** polyclonal antibody from Santa Cruz, **C** monoclonal antibody from R&D Systems, and **D** polyclonal antibody from R&D Systems. In this study, a monoclonal antibody (Santa Cruz) was used to determine the expression level of the MUPs.

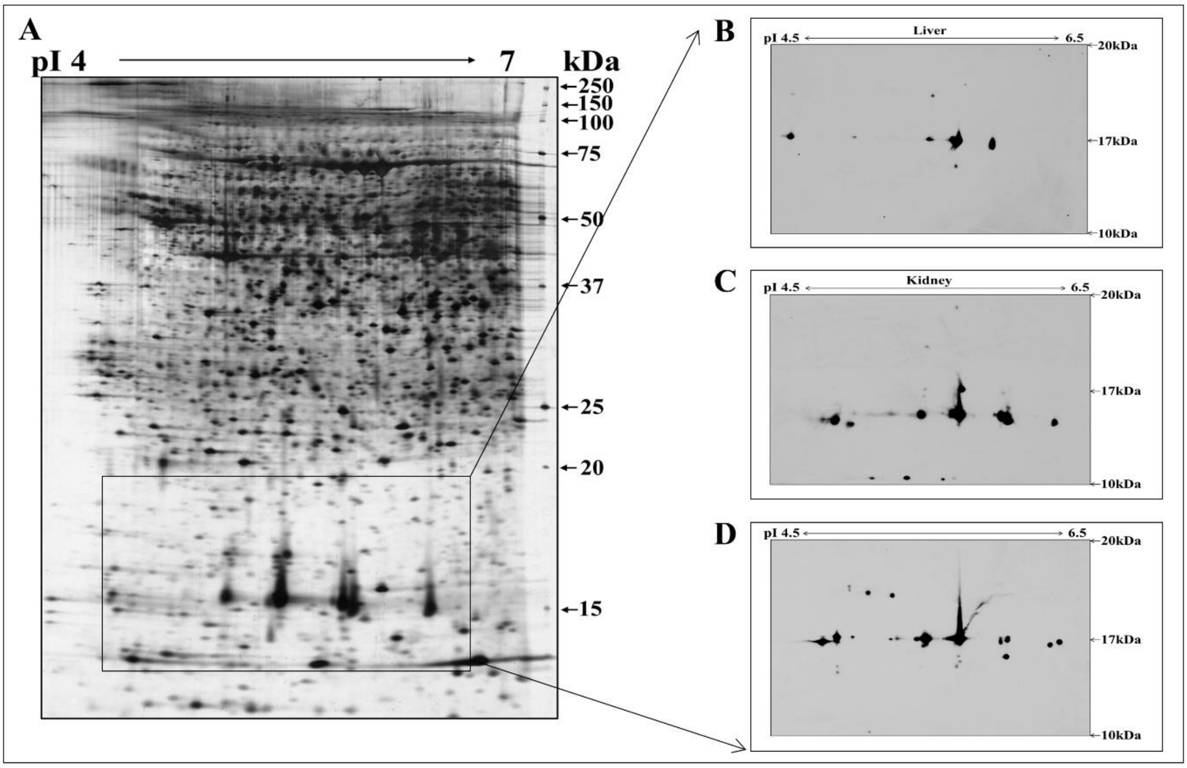

Fig. S6 2-DE immunoblot analysis of MUP isoforms in the liver, kidneys, and urine. The 2-DE gel in the pI area from 4.5 to 6.5 and molecular weight range from 10 kDa to 20 kDa was removed and electroblotted onto PVDF membranes.

A

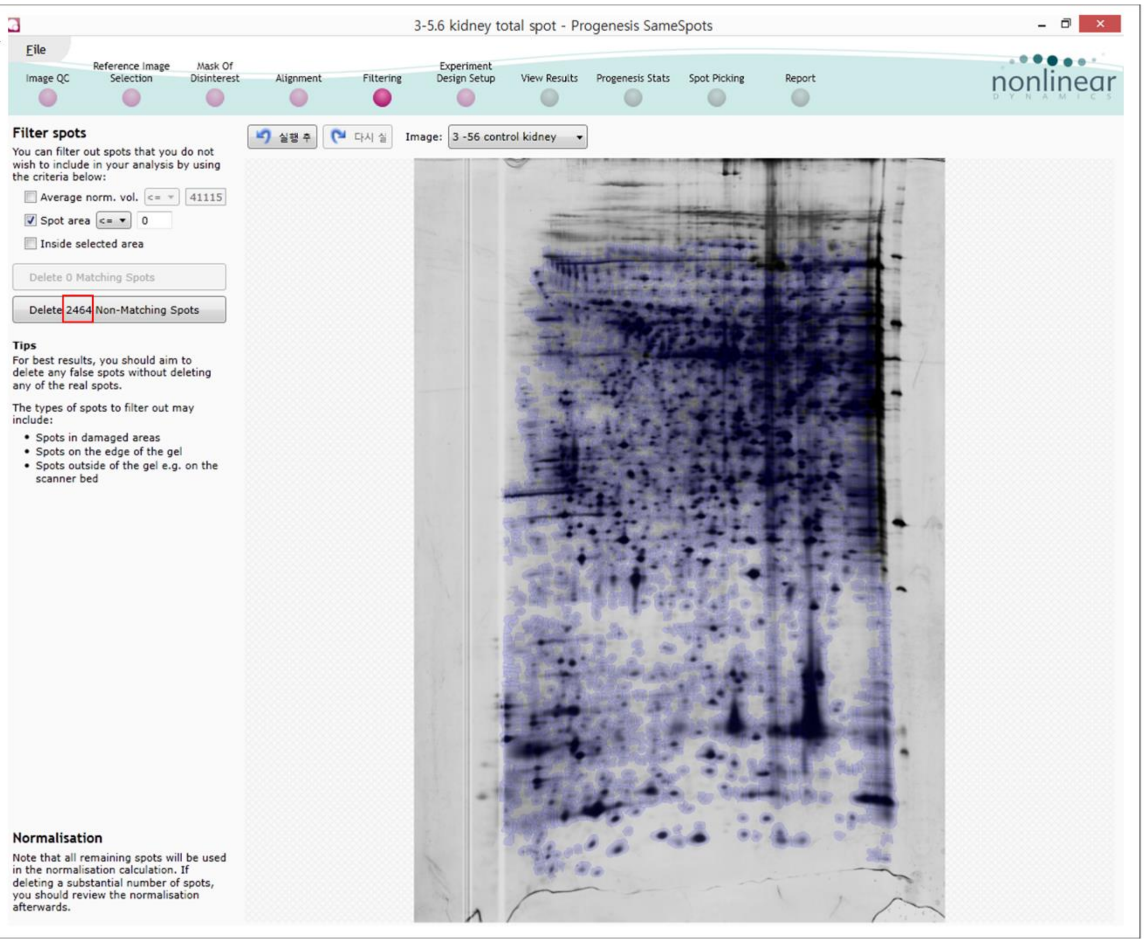

3-5.6 kidney total spot - Progenesis SameSpots
File
Image QC
Reference Image Selection
Mask Of Disinterest
Alignment
Filtering
Experiment Design Setup
View Results
Progenesis Stats
Spot Picking
Report
nonlinear
DYNAMICS
Filter spots
You can filter out spots that you do not wish to include in your analysis by using the criteria below:
Average norm. vol.
<=
41115
Spot area
<=
0
Inside selected area
Delete 0 Matching Spots
Delete 2464 Non-Matching Spots
Tips
For best results, you should aim to delete any false spots without deleting any of the real spots.
The types of spots to filter out may include:
Spots in damaged areas
Spots on the edge of the gel
Spots outside of the gel e.g. on the scanner bed
Normalisation
Note that all remaining spots will be used in the normalisation calculation. If deleting a substantial number of spots, you should review the normalisation afterwards.
Image:
3 -56 control kidney

B

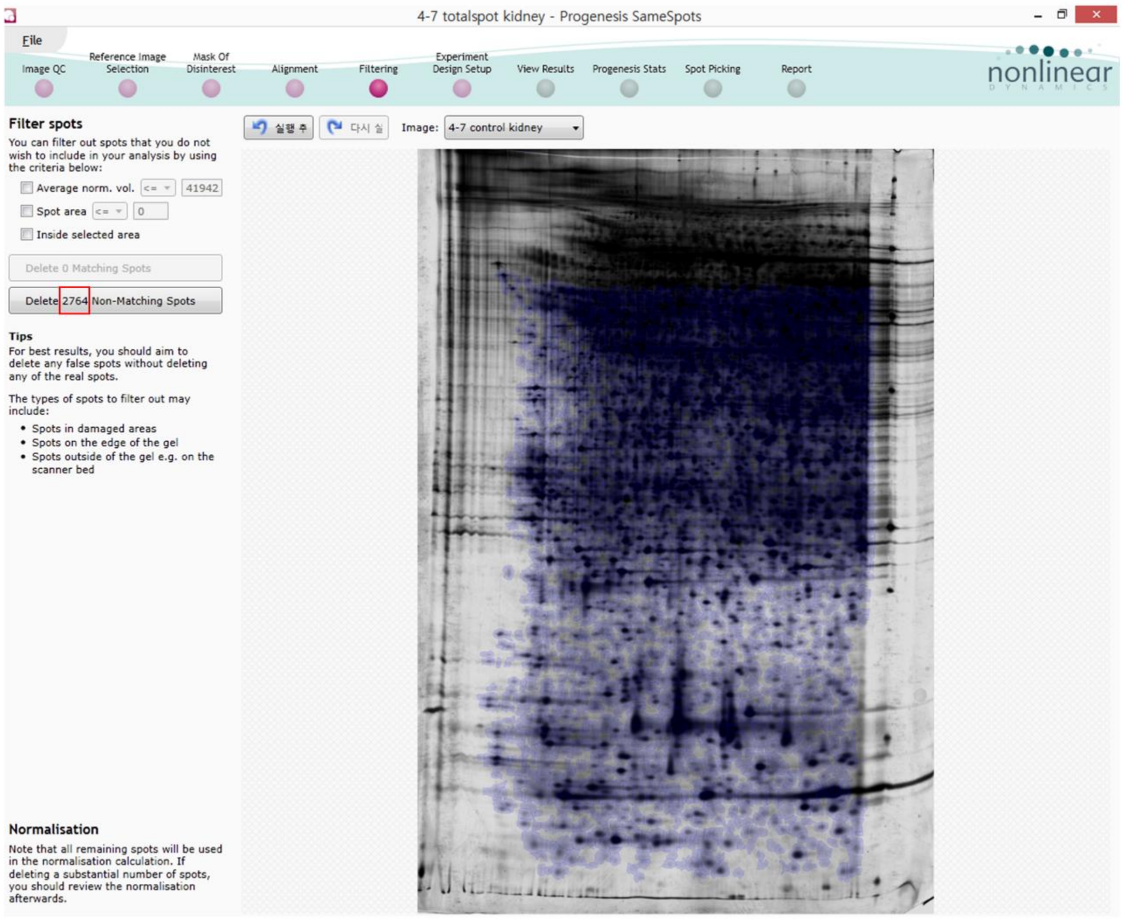
4-7 totalspot kidney - Progenesis SameSpots
File
Image QC
Reference Image Selection
Mask Of Disinterest
Alignment
Filtering
Experiment Design Setup
View Results
Progenesis Stats
Spot Picking
Report
nonlinear
DYNAMICS
Filter spots
You can filter out spots that you do not wish to include in your analysis by using the criteria below:
Average norm. vol.
<=
41942
Spot area
<=
0
Inside selected area
Delete 0 Matching Spots
Delete 2764 Non-Matching Spots
Tips
For best results, you should aim to delete any false spots without deleting any of the real spots.
The types of spots to filter out may include:
Spots in damaged areas
Spots on the edge of the gel
Spots outside of the gel e.g. on the scanner bed
Normalisation
Note that all remaining spots will be used in the normalisation calculation. If deleting a substantial number of spots, you should review the normalisation afterwards.
Image:
4-7 control kidney

C

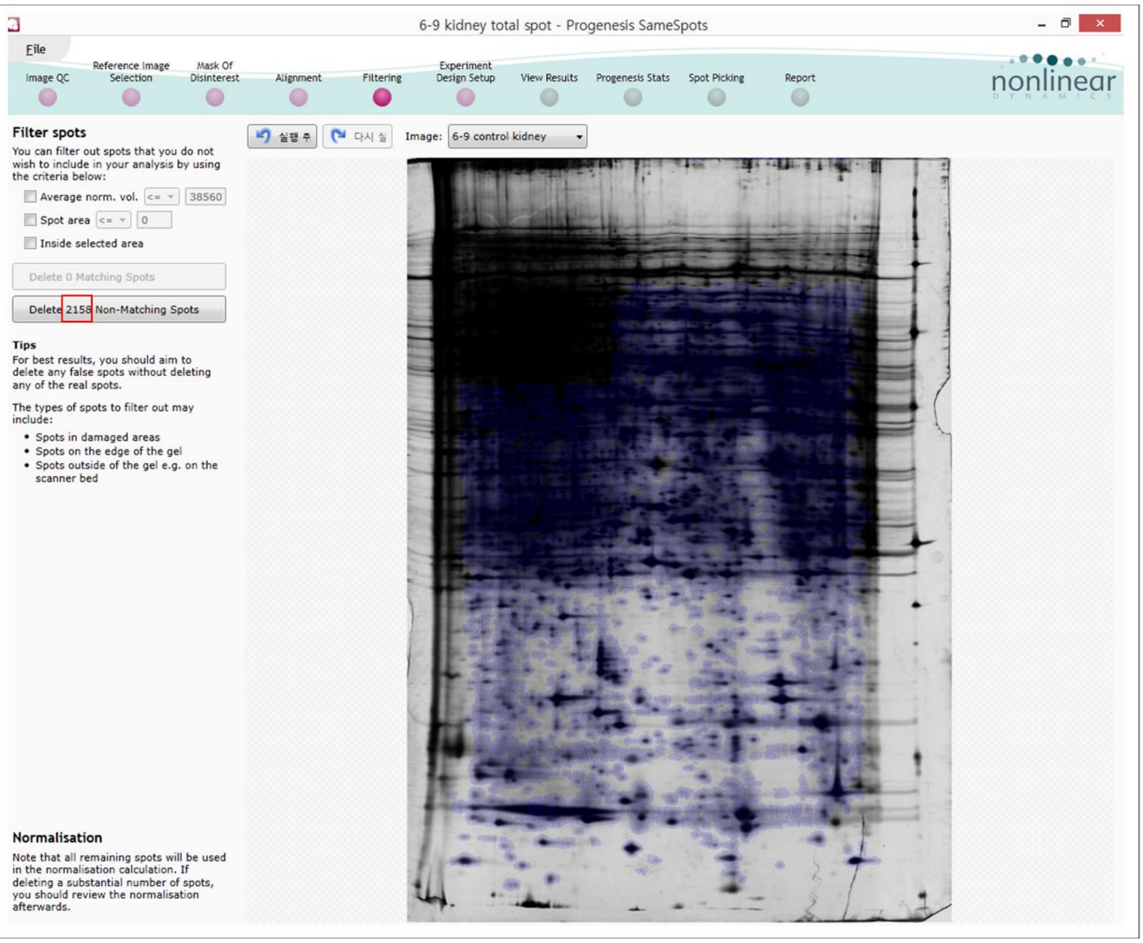

6-9 kidney total spot - Progenesis SameSpots
File
Image QC
Reference Image Selection
Mask Of Disinterest
Alignment
Filtering
Experiment Design Setup
View Results
Progenesis Stats
Spot Picking
Report
nonlinear
DYNAMICS
Filter spots
You can filter out spots that you do not wish to include in your analysis by using the criteria below:
Average norm. vol. <= 38560
Spot area <= 0
Inside selected area
Delete 0 Matching Spots
Delete 2158 Non-Matching Spots
Tips
For best results, you should aim to delete any false spots without deleting any of the real spots.
The types of spots to filter out may include:
Spots in damaged areas
Spots on the edge of the gel
Spots outside of the gel e.g. on the scanner bed
Normalisation
Note that all remaining spots will be used in the normalisation calculation. If deleting a substantial number of spots, you should review the normalisation afterwards.
Image: 6-9 control kidney

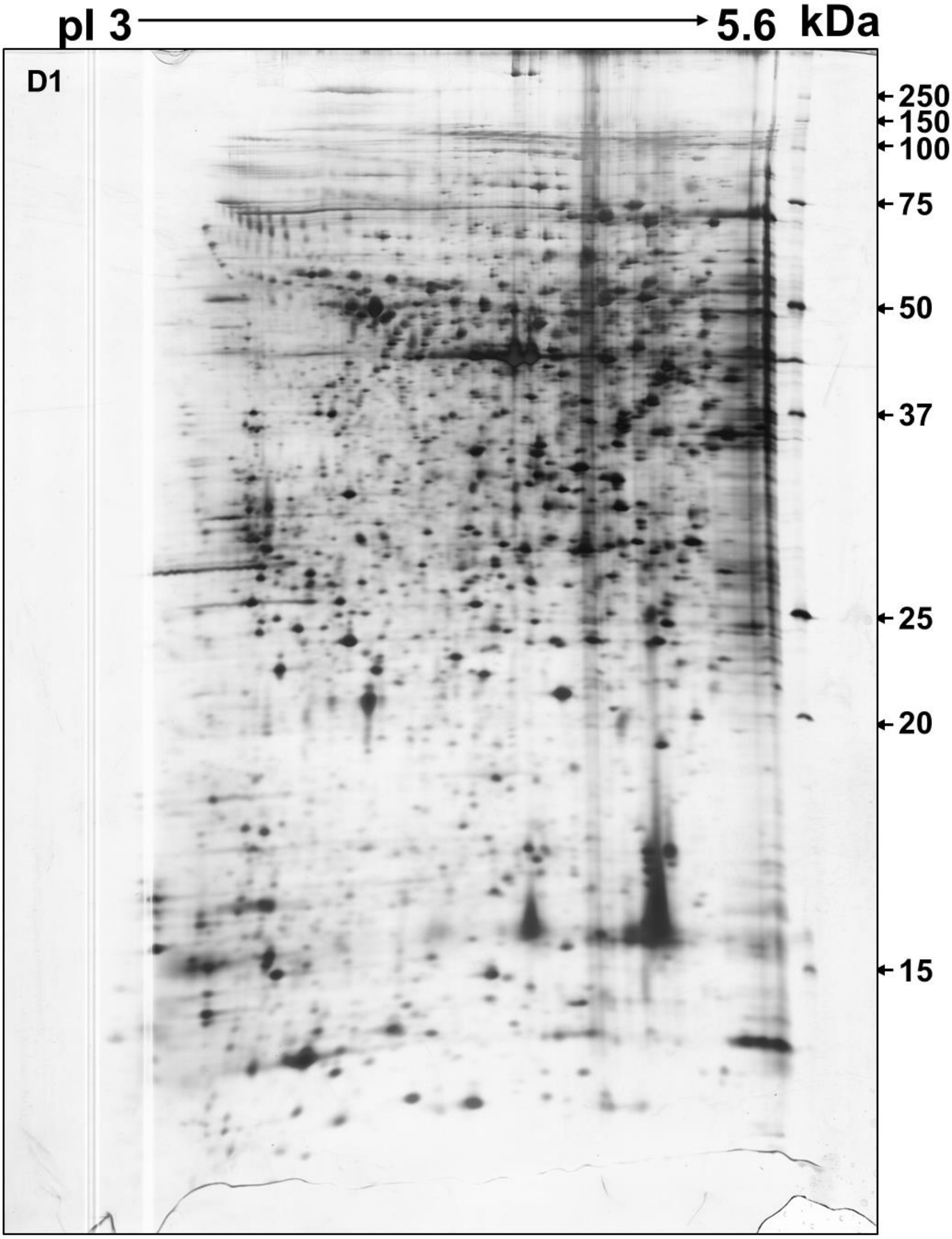
pI 3
5.6
kDa
D1
250
150
100
75
50
37
25
20
15

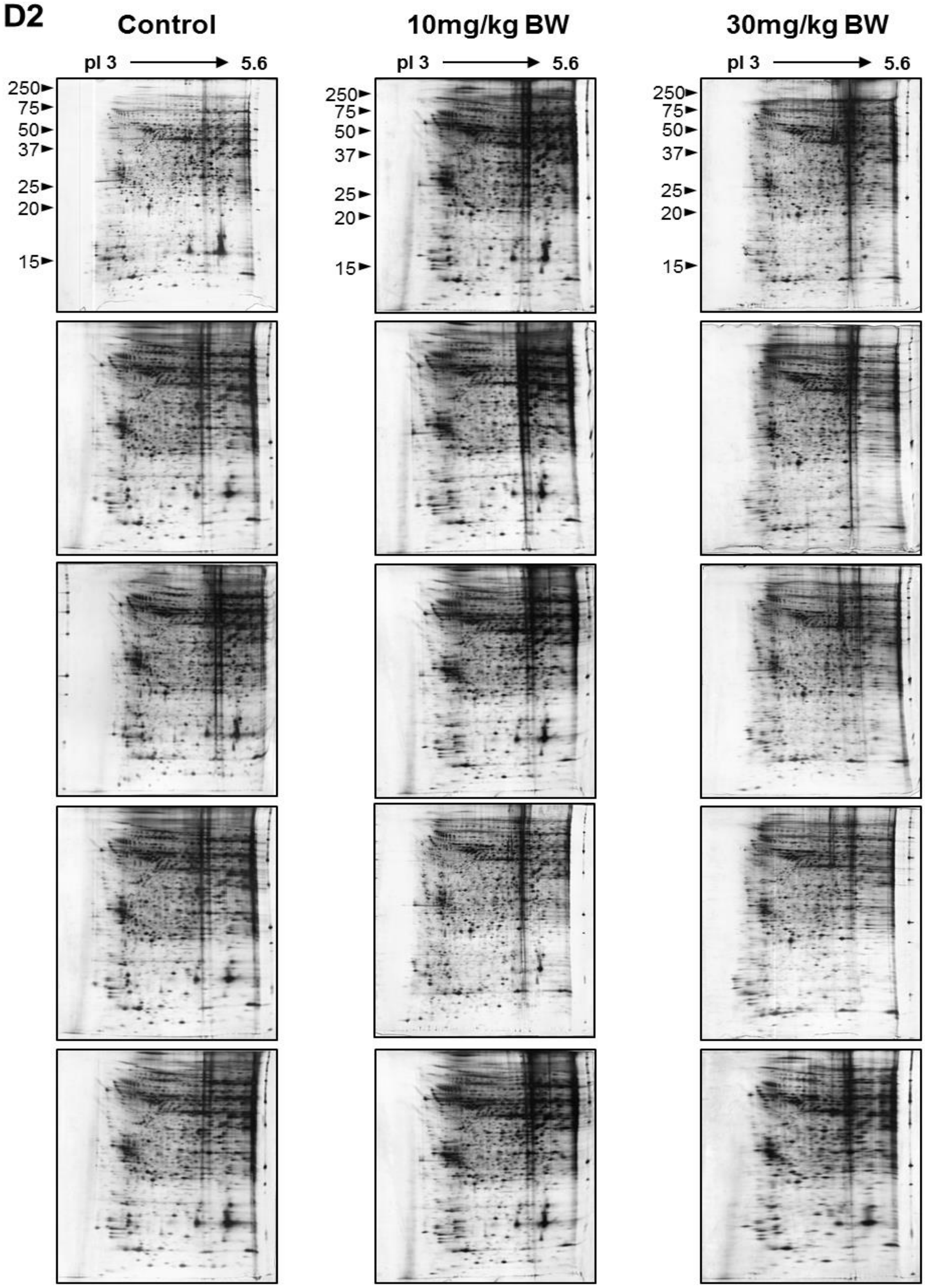
D2
Control
10mg/kg BW
30mg/kg BW
pI 3
5.6
250
75
50
37
25
20
15

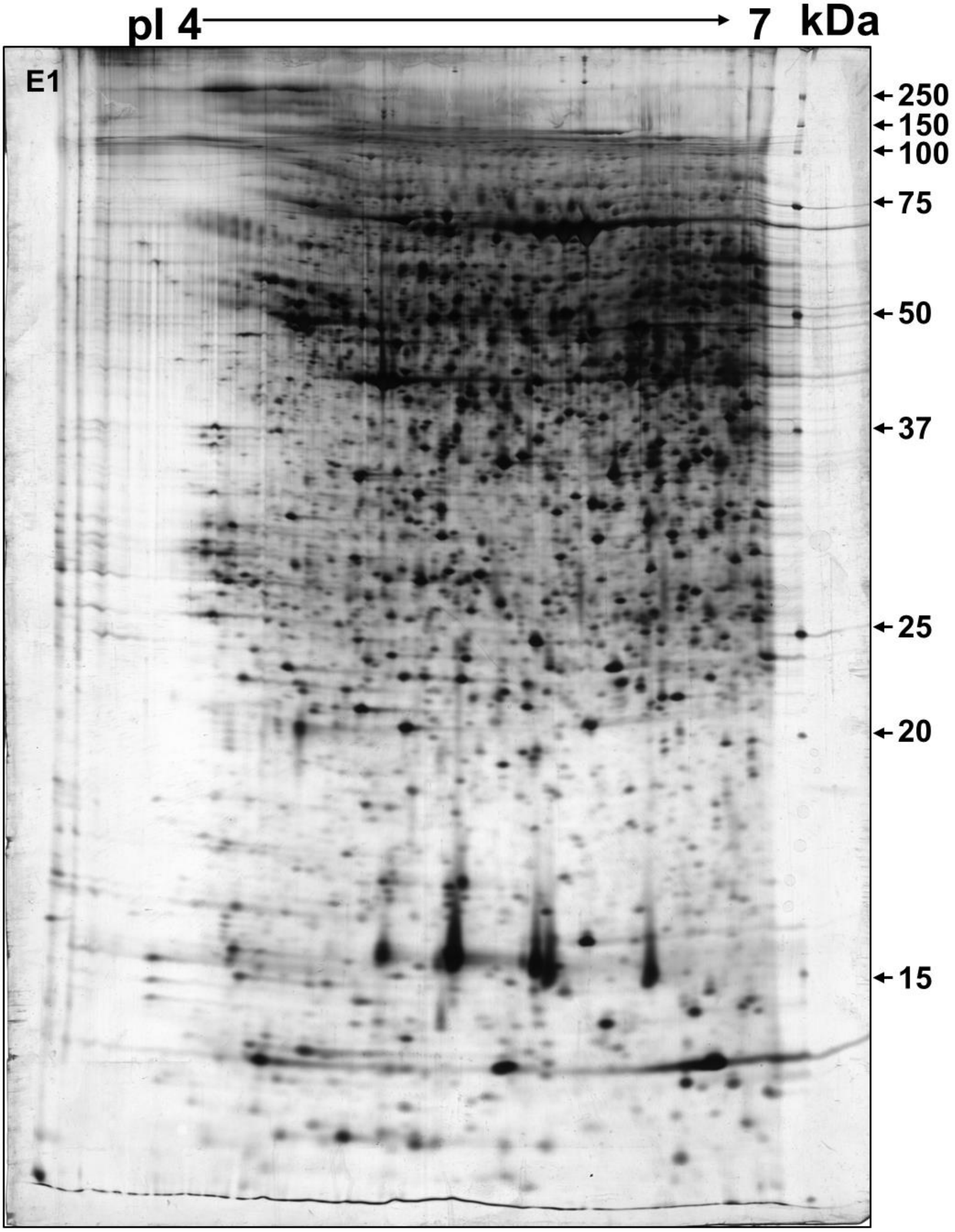
pI 4
7
kDa
E1
250
150
100
75
50
37
25
20
15

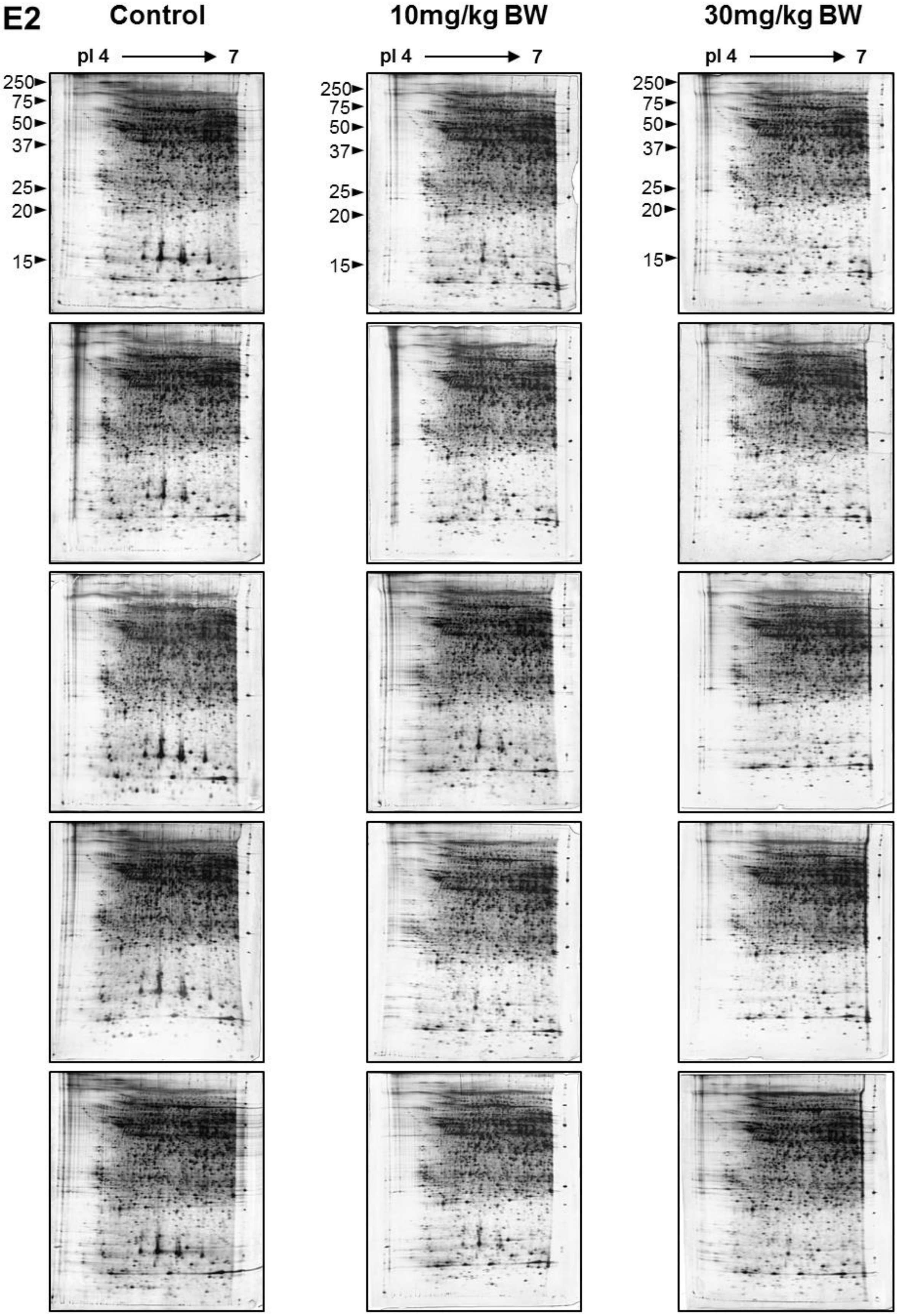
E2
Control
10mg/kg BW
30mg/kg BW
pI 4
7
250
75
50
37
25
20
15

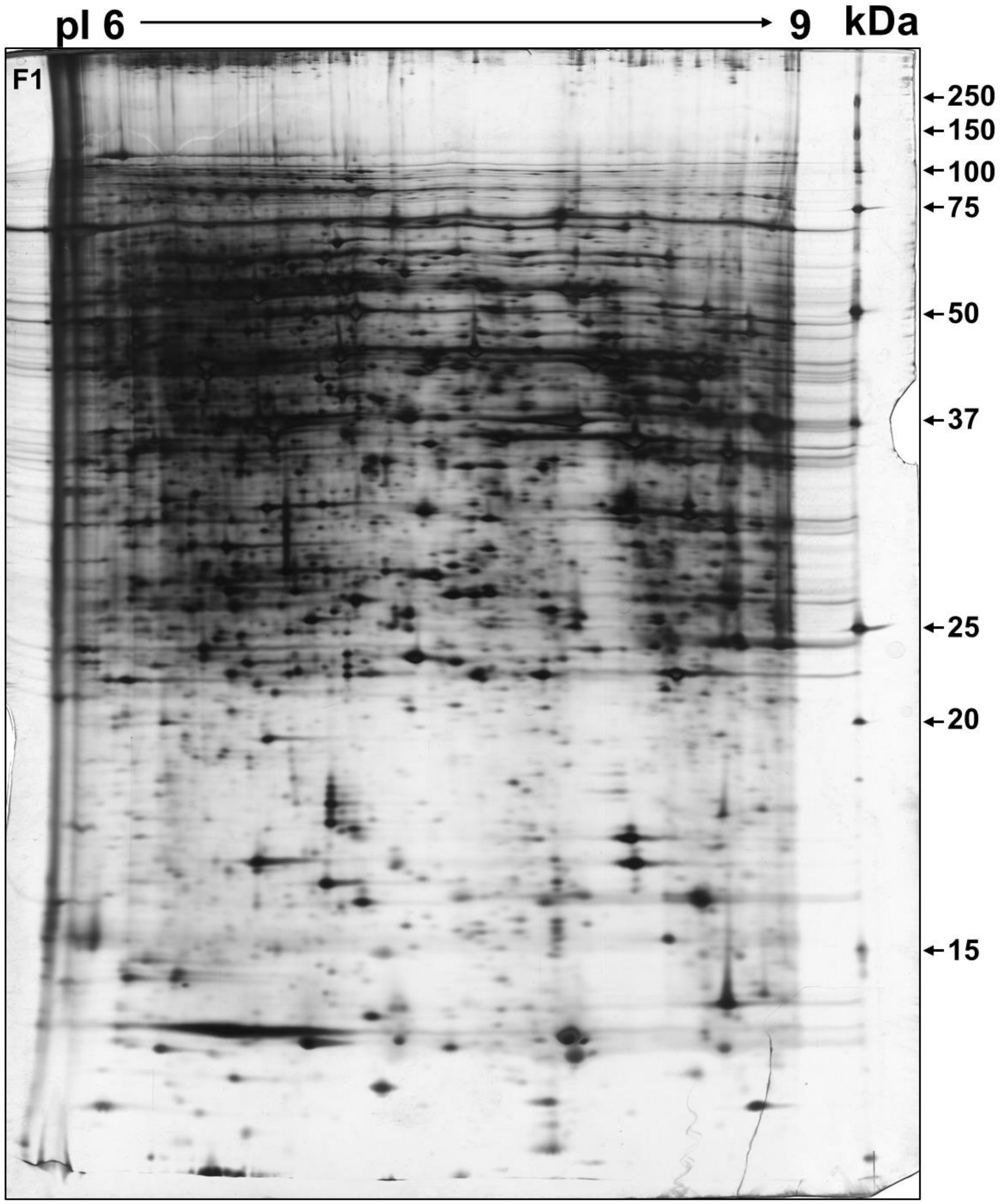
pI 6
9
kDa
F1
250
150
100
75
50
37
25
20
15

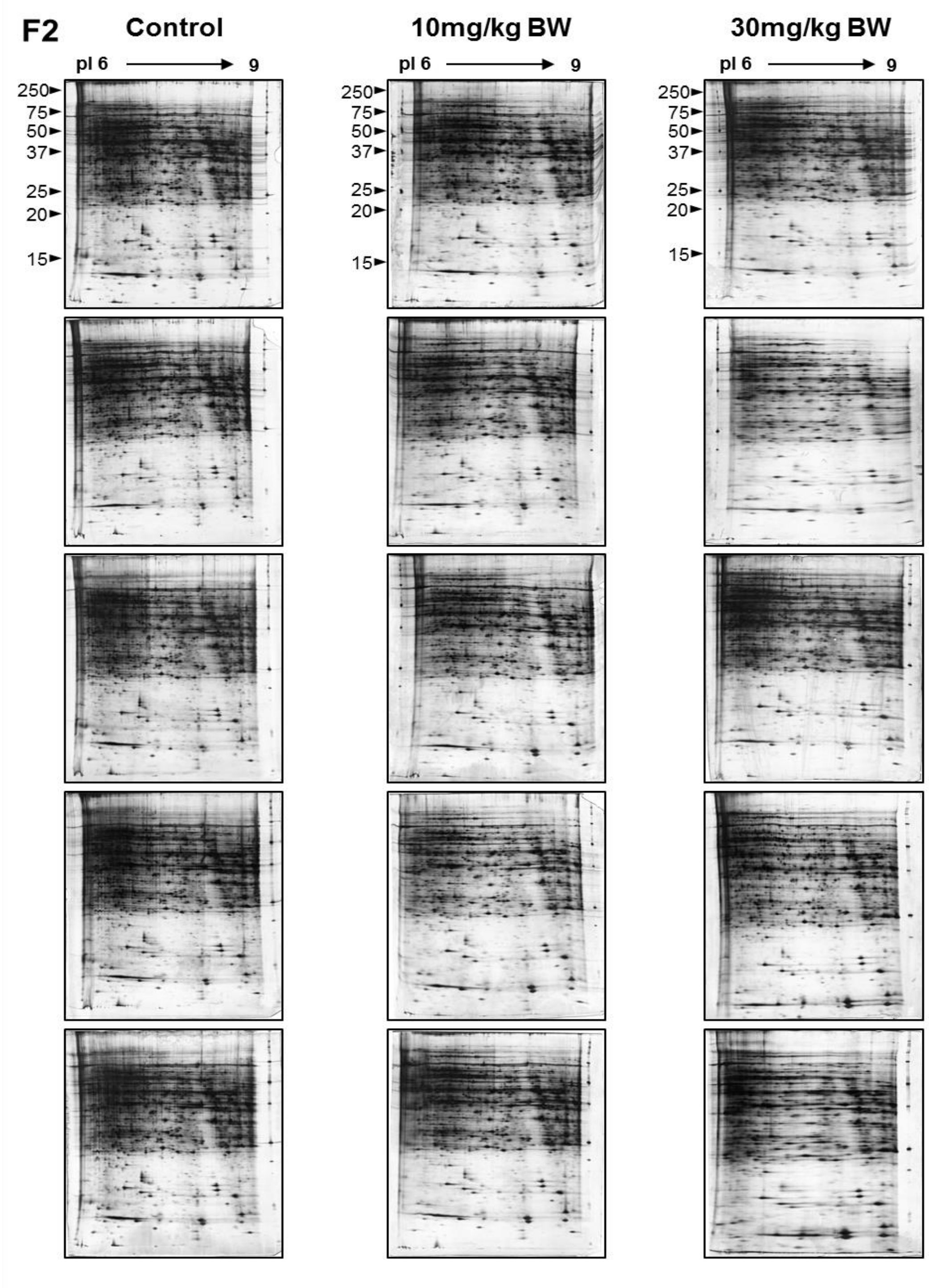

Supplementary image and original gel data: Gel image data, After separation on SDS-PAGE gels, the proteins were visualized by silver staining. The silver-stained gels were scanned using a 3600 × 4900 dpi instrument and the image files were transformed into a TIFF format with linear grayscale values. The computer analysis of the 2DE-image was carried out using Progenesis Samespot image analysis software. Three different ranges of pI strips (3-5.6, 4-7, and 6-9), showed 2464 (**A**), 2764 (**B**), and 2158 (**C**) protein spots present in the gels, respectively. **Original gel data**, (**D1/2**) Original gels (pI 3-5.6), (**E1/2**) Original gels (pI 4-7), (**F1/2**) Original gels (pI 6-9).

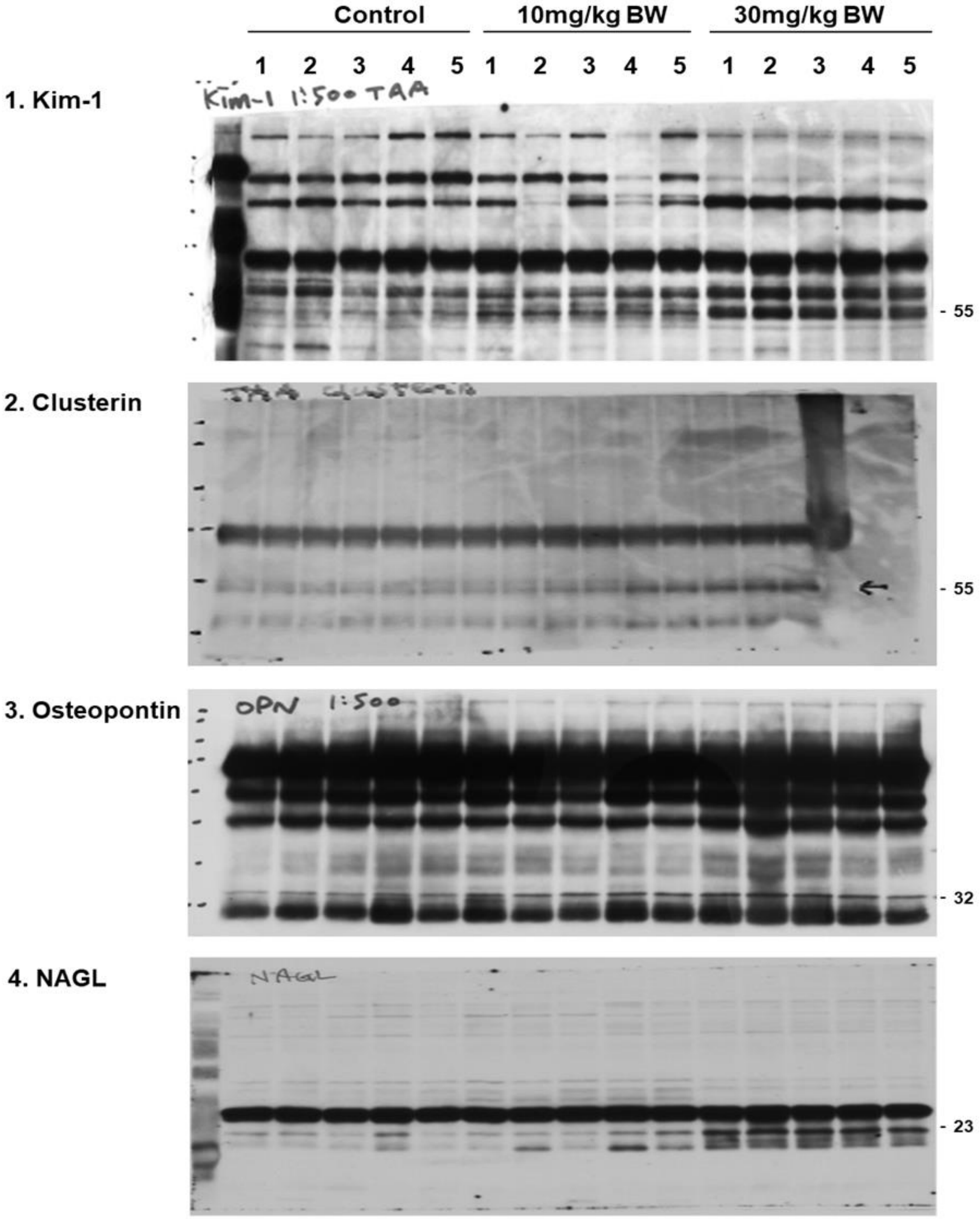
Control
10mg/kg BW
30mg/kg BW
1 2 3 4 5 1 2 3 4 5 1 2 3 4 5
1. Kim-1
Kim-1 1:500 TAA
- 55
2. Clusterin
- 55
3. Osteopontin
OPN 1:500
- 32
4. NAGL
NAGL
- 23

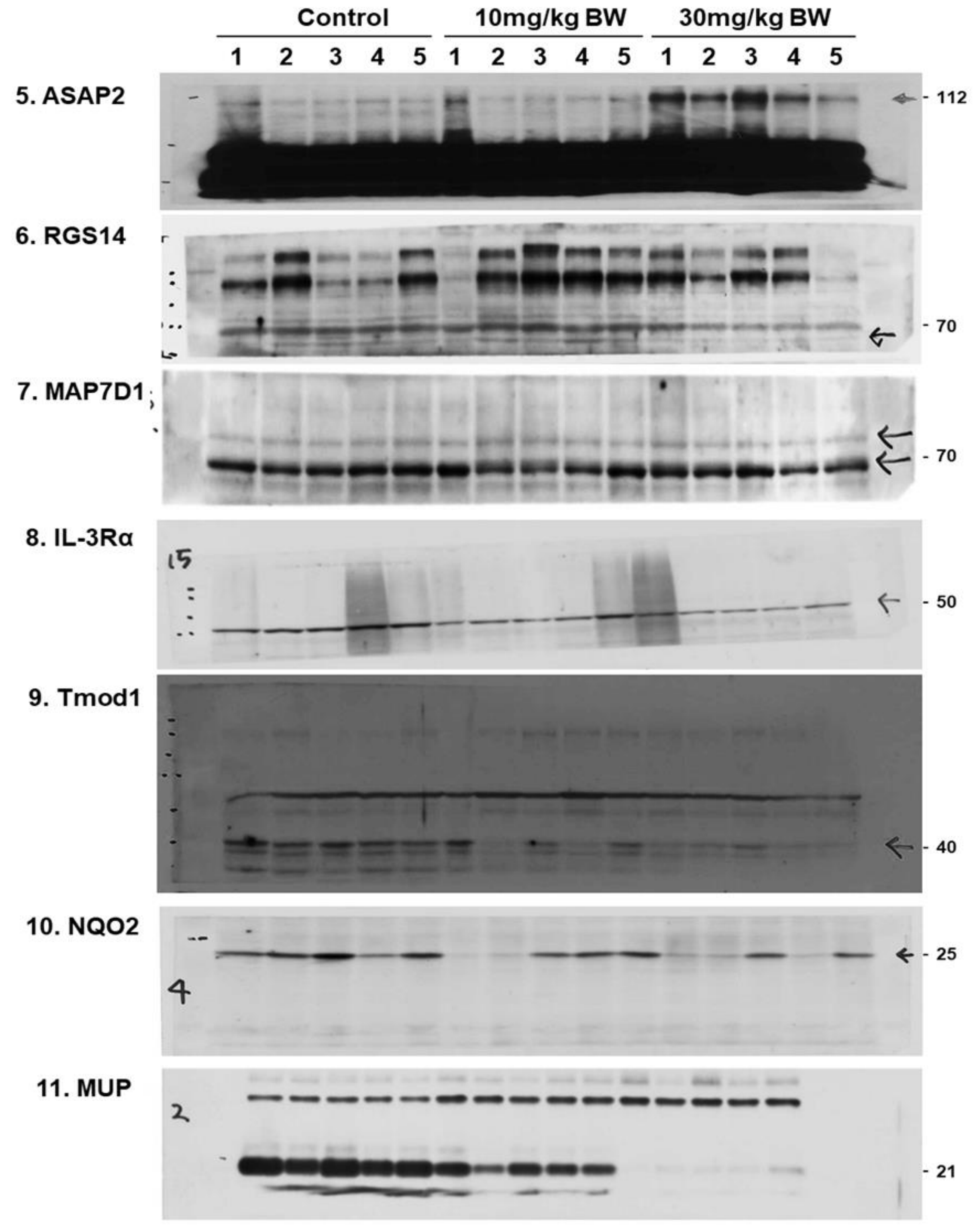

Supplementary Western blot data: Original western blots, **(1)** Kim-1, **(2)** Clusterin, **(3)** Osteopontin, **(4)** NGAL, **(5)** ASAP2, **(6)** RGS14, **(7)** MAP7D1, **(8)** IL-3Rα, **(9)** Tmod1, **(10)** NQO2.

Supplementary protein identification data

Spot No.	Accession	Description	mW (Da)	pI (pH)	Peptides	Theoretical Peptides	Coverage (%)	Products	Products RMS Mass Error (ppm)	Products RMS RT Error (min)	Protein ID
1	F1LZT6	Protein Asap2 Fragment	103093	6.3633	9	75	7.4595	49	27.3644	0.0234432	26089
Precursor MH+ (Da)	z	Peptide MH+ (Da)	Score	Start	End	Sequence	Retention Time (min)	Products	Products RMS Mass Error (ppm)	Products RMS RT Error (min)	Protein ID
973.5304	1.66	973.5313	6.4967	393	400	(K)EIISEVQR(M)	25.5402	19	21.5198	0.0142	26089,
821.5088	1	821.4767	5.3408	75	81	(K)ELTALFK(N)	55.6755	6	37.1421	0.0365	26089,
2043.0387	2	2043.0604	5.1768	614	633	(R)GKASIEIANESGETPLDIAK(R)	37.9571	4	19.545	0.0274	26089,
3838.9163	3.4	3838.947	5.1391	687	720	(R)REDRPVSFYQLGSSQFQPNAVSLARDTANLTKDK(Q)	36.5406	10	22.7439	0.0176	26089,
579.3508	1	579.3501	0	77	81	(L)TALFK(N)	53.9599	2	39.9053	0.0421	26089,
554.2834	1	554.2821	0	393	397	(K)EIISE(V)	25.5277	4	10.2295	0.0156	26089,
618.3287	1	618.3206	0	396	400	(I)SEVQR(M)	24.5575	1	4.263	0.001	26089,
840.4348	1	840.4322	0	687	693	(R)REDRPVS(F)	36.2709	2	49.8765	0.0353	26089,
561.3411	1	561.3395	0	77	81	(L)TALFK(N)	52.8756	1	65.7091	0.0441	26089,

Spot No.	Accession	Description	mW (Da)	pI (pH)	Peptides	Theoretical Peptides	Coverage (%)	Products	Products RMS Mass Error (ppm)	Products RMS RT Error (min)	Protein ID
2	G3V6P6	Putative RNA binding protein 3	16972	8.8901	8	13	21.7949	106	17.7032	0.0121813	8025
Precursor MH+ (Da)	z	Peptide MH+ (Da)	Score	Start	End	Sequence	Retention Time (min)	Products	Products RMS Mass Error (ppm)	Products RMS RT Error (min)	Protein ID
1997.9299	2.57	1997.9174	8.0722	48	65	(R)GFGFITFTNPEHASDAMR(A)	37.8824	27	15.3498	0.01	8025,
1567.6984	2.32	1567.6924	8.0476	117	130	(R)YDSRPGGYGYGYGR(S)	20.7355	18	13.8627	0.0123	8025,
2013.9315	2.61	2013.9124	7.8268	48	65	(R)GFGFITFTNPEHASDAMR(A)	36.5173	23	13.0112	0.0125	8025,
1810.826	2	1810.8256	6.58	117	132	(R)YDSRPGGYGYGYGRSR(D)	31.937	5	35.5232	0.016	8025,
1391.6173	2	1391.6008	0	54	65	(T)FTNPEHASDAMR(A)	36.5233	7	25.6891	0.0217	8025,
1404.6383	2	1404.6292	0	118	130	(Y)DSRPGGYGYGYGR(S)	20.7395	10	8.324	0.0071	8025,
1289.5997	2	1289.6022	0	119	130	(D)SRPGGYGYGYGR(S)	20.7377	13	20.17	0.0082	8025,
1752.7806	2	1752.801	0	51	65	(G)FITFTNPEHASDAMR(A)	34.4319	3	29.4143	0.0153	8025,

Spot No.	Accession	Description	mW (Da)	pI (pH)	Peptides	Theoretical Peptides	Coverage (%)	Products	Products RMS Mass Error (ppm)	Products RMS RT Error (min)	Protein ID
3	D4ACM1	Protein Elp3	64030	8.9312	4	52	8.4806	37	42.1688	0.0142513	24363
Precursor MH+ (Da)	z	Peptide MH+ (Da)	Score	Start	End	Sequence	Retention Time (min)	Products	Products RMS Mass Error (ppm)	Products RMS RT Error (min)	Protein ID
1120.5575	1.66	1120.5602	5.9635	410	418	(R)MKDLGIQCR(D)	33.2778	17	41.7241	0.0151	24363,
1695.8033	2	1695.816	4.8837	285	299	(K)AVCESFHLAKDSGFK(V)	33.582	5	54.6599	0.012	24363,
1055.4995	2	1055.5156	4.7655	161	168	(R)YDPYLQTR(H)	20.4538	6	31.0795	0.0121	24363,
1922.9233	2	1922.8888	4.6626	511	526	(K)FQHQGFGMLLMEEAER(I)	33.2761	9	41.4516	0.0151	24363,

Spot No.	Accession	Description	mW (Da)	pI (pH)	Peptides	Theoretical Peptides	Coverage (%)	Products	Products RMS Mass Error (ppm)	Products RMS RT Error (min)	Protein ID
4	P70567	Tropomodulin 1	40454	4.7827	3	30	11.6992	23	33.6454	0.0266428	7179
Precursor MH+ (Da)	z	Peptide MH+ (Da)	Score	Start	End	Sequence	Retention Time (min)	Products	Products RMS Mass Error (ppm)	Products RMS RT Error (min)	Protein ID
3338.7942	3.51	3338.7666	6.1143	256	286	(K)TLNVESNFISGAGILCLVEALPHNTSLVELK(I)	41.7671	15	30.3	0.0283	7179,
1213.5599	1	1213.6171	4.6624	287	297	(K)IDNQSQPLGNK(V)	40.1375	1	0	0	7179,
3320.7273	3.63	3320.7559	0	256	286	(K)TLNVESNFISGAGILCLVEALPHNTSLVELK(I)	41.3309	7	41.8446	0.0235	7179,

Spot No.	Accession	Description	mW (Da)	pI (pH)	Peptides	Theoretical Peptides	Coverage (%)	Products	Products RMS Mass Error (ppm)	Products RMS RT Error (min)	Protein ID
5	P11598	Protein disulfide isomerase A3	56587	5.8081	16	53	15.8416	93	24.2949	0.0177072	5086
Precursor MH+ (Da)	z	Peptide MH+ (Da)	Score	Start	End	Sequence	Retention Time (min)	Products	Products RMS Mass Error (ppm)	Products RMS RT Error (min)	Protein ID
1191.6031	1.95	1191.6003	7.2716	63	73	(R)LAPEYEAAATR(L)	20.9244	17	15.4712	0.0154	5086,
1236.519	2	1236.5127	6.7529	108	119	(R)DGEEAGAYDGPR(T)	17.938	12	17.7535	0.0137	5086,
1394.6642	2	1394.6587	6.4327	162	173	(R)DLFSDGHSEFLK(A)	32.9523	6	13.6376	0.0118	5086,
1040.5736	2	1040.5735	6.432	120	129	(R)TADGIVSHLK(K)	22.1801	6	15.5233	0.0267	5086,
995.562	2	995.5633	6.2264	131	140	(K)QAGPASVPLR(T)	21.6059	5	17.3409	0.0128	5086,
697.4506	1	697.4607	6.1223	76	82	(K)GIVPLAK(V)	24.4105	5	37.5609	0.0198	5086,
997.4992	1	997.5102	5.8393	153	161	(K)DASVVGFFR(D)	35.3818	6	33.3379	0.0197	5086,
1125.5435	1.95	1125.5436	5.736	272	280	(K)NTKGSNYWR(N)	18.7078	9	47.4995	0.0176	5086,
489.2784	1	489.278	0	69	73	(E)AAATR(L)	20.0598	1	6.9102	0.0287	5086,
735.3462	1	735.342	0	113	119	(A)GAYDGPR(T)	17.942	2	25.8582	0.0164	5086,
977.554	2	977.5527	0	131	140	(K)QAGPASVPLR(T)	21.608	0	0	0	5086,
571.3621	1	571.3562	0	136	140	(A)SVPLR(T)	20.4616	1	5.0115	0.0251	5086,
1166.549	2	1166.5477	0	164	173	(L)FSDGHSEFLK(A)	32.9512	4	10.4391	0.0134	5086,
1019.4833	2	1019.4793	0	165	173	(F)SDGHSEFLK(A)	32.9553	3	12.0536	0.0193	5086,
1107.5269	2	1107.533	0	272	280	(K)NTKGSNYWR(N)	20.1066	2	29.9011	0.0415	5086,
1007.4789	1.93	1007.4793	0	65	73	(A)PEYEAAATR(L)	20.925	14	18.6652	0.0136	5086,

Spot No.	Accession	Description	mW (Da)	pI (pH)	Peptides	Theoretical Peptides	Coverage (%)	Products	Products RMS Mass Error (ppm)	Products RMS RT Error (min)	Protein ID
6	P62749	Hippocalcin like protein 1	22324	5.1621	7	20	32.1244	42	26.3681	0.0194005	3144
Precursor MH+ (Da)	z	Peptide MH+ (Da)	Score	Start	End	Sequence	Retention Time (min)	Products	Products RMS Mass Error (ppm)	Products RMS RT Error (min)	Protein ID
1238.726	2	1238.7216	7.0687	8	17	(K)LRPEVLQDLR(E)	28.719	9	11.9178	0.0116	3144,
905.4631	1.81	905.4628	6.6955	64	70	(K)FAEHVFR(T)	22.3436	8	30.577	0.0268	3144,
1492.7166	2	1492.7107	6.5812	51	63	(K)IYANFFPYGDASK(F)	35.5072	5	14.5825	0.0154	3144,
1488.6693	1.97	1488.6602	6.4348	71	83	(R)TFDTNSDGTIDFR(E)	29.1183	7	8.2885	0.0148	3144,
1352.6262	1.76	1352.6151	6.3447	182	193	(R)LLQCDPSSASQF(-)	28.7625	6	49.7483	0.0188	3144,
781.4269	1	781.3947	5.7171	131	137	(K)MVSSVMK(M)	34.5704	4	0	0	3144,
595.3518	1	595.3562	0	8	12	(K)LRPEV(L)	28.7264	3	14.8095	0.0159	3144,

Spot No.	Accession	Description	mW (Da)	pI (pH)	Peptides	Theoretical Peptides	Coverage (%)	Products	Products RMS Mass Error (ppm)	Products RMS RT Error (min)	Protein ID
7	P14942	Glutathione S transferase alpha 4	25493	7.2964	11	20	23.4234	63	22.7112	0.0207633	2942
Precursor MH+ (Da)	z	Peptide MH+ (Da)	Score	Start	End	Sequence	Retention Time (min)	Products	Products RMS Mass Error (ppm)	Products RMS RT Error (min)	Protein ID
949.568	1.18	949.5717	7.3471	70	78	(R)AILSYLAAK(Y)	32.5032	13	30.8318	0.0167	2942,
929.4763	1.24	929.4767	7.2466	132	138	(R)YFPVFEK(I)	32.5021	9	23.5874	0.0116	2942,
1478.7814	2.52	1478.775	7.2104	205	217	(R)KPPPDGHYVDVVR(T)	21.0223	12	16.7153	0.033	2942,
946.4784	1.35	946.4781	6.7374	7	13	(K)LYYFQGR(G)	27.5205	4	7.9051	0.0113	2942,
932.4926	1.54	932.4948	6.5714	197	204	(K)FLQPGSQR(K)	19.3413	5	8.6738	0.0107	2942,
885.5406	1.49	885.5404	6.5675	188	195	(R)ISNIPTIK(K)	25.541	5	10.8272	0.0128	2942,
672.3422	1	672.3424	0	199	204	(L)QPGSQR(K)	19.3457	4	14.2708	0.0224	2942,
544.2862	1	544.2838	0	200	204	(Q)PGSQR(K)	19.336	4	23.9526	0.0239	2942,
1350.6846	2	1350.68	0	206	217	(K)PPPDGHYVDVVR(T)	19.3905	1	14.8742	0.0019	2942,
1253.6355	2	1253.6273	0	207	217	(P)PPDGHYVDVVR(T)	23.5318	3	48.0866	0.03	2942,
685.4204	1	685.4243	0	190	195	(S)NIPTIK(K)	25.5497	3	9.9551	0.0102	2942,

Spot No.	Accession	Description	mW (Da)	pI (pH)	Peptides	Theoretical Peptides	Coverage (%)	Products	Products RMS Mass Error (ppm)	Products RMS RT Error (min)	Protein ID
8	F1M0B2	Uncharacterized protein Fragment	57714	7.8838	18	41	11.5888	97	21.3757	0.015062	20995
**Precursor MH+ (Da)**	**z**	**Peptide MH+ (Da)**	**Score**	**Start**	**End**	**Sequence**	**Retention Time (min)**	**Products**	**Products RMS Mass Error (ppm)**	**Products RMS RT Error (min)**	**Protein ID**
1302.7285	1.93	1302.7151	7.4345	299	310	(R)SLDLDSIIAEVK(A)	42.1666	24	9.1118	0.0139	20995,
1179.6143	1.94	1179.6003	7.2131	332	341	(K)YEELQITAGR(H)	26.5065	19	10.1715	0.0135	20995,
827.4305	1.26	827.4298	6.5623	153	159	(K)FASFIDK(V)	28.0801	4	5.5398	0.0096	20995,
1115.5874	2	1115.5944	5.587	399	408	(K)LEGLEDALQK(S)	18.4311	3	38.1461	0.0206	20995,
2823.3357	3.51	2823.3916	5.4547	174	196	(K)CALLTRRPDLEPMFEDYINNLSR(R)	31.9101	5	58.7143	0.0281	20995,
609.3263	1	609.3243	0	155	159	(A)SFIDK(V)	28.0884	4	34.1383	0.0198	20995,
744.3797	1	744.3774	0	299	305	(R)SLDLDSI(I)	42.1645	3	13.4964	0.0063	20995,
1156.6147	1	1156.6096	0	299	309	(R)SLDLDSIIAEV(K)	42.1591	6	22.4108	0.0107	20995,
1284.7163	2	1284.7046	0	299	310	(R)SLDLDSIIAEVK(A)	40.7176	3	21.3502	0.0155	20995,
874.4938	1	874.488	0	303	310	(L)DSIIAEVK(A)	42.1545	5	6.0672	0.0122	20995,
559.3491	1	559.345	0	306	310	(I)IAEVK(A)	42.1517	2	15.2283	0.0105	20995,
446.2651	1	446.2609	0	307	310	(I)AEVK(A)	42.1533	1	4.104	0.0308	20995,
663.3035	1	663.2984	0	332	336	(K)YEELQ(I)	26.5144	1	0	0	20995,
1161.596	2	1161.5898	0	332	341	(K)YEELQITAGR(H)	26.5042	0	0	0	20995,
758.4469	1	758.4519	0	335	341	(E)LQITAGR(H)	26.5005	5	5.9466	0.0127	20995,
517.313	1	517.3093	0	337	341	(Q)ITAGR(H)	26.505	3	16.8092	0.0192	20995,
404.2266	1	404.2252	0	338	341	(I)TAGR(H)	26.5108	3	30.068	0.0068	20995,
680.3622	1	680.3614	0	154	159	(F)ASFIDK(V)	28.0809	6	27.757	0.0086	20995,

Spot No.	Accession	Description	mW (Da)	pI (pH)	Peptides	Theoretical Peptides	Coverage (%)	Products	Products RMS Mass Error (ppm)	Products RMS RT Error (min)	Protein ID
9	P02761	Major urinary protein	20723	5.7964	24	20	55.2486	192	20.0973	0.0209221	4360
**Precursor MH+ (Da)**	**z**	**Peptide MH+ (Da)**	**Score**	**Start**	**End**	**Sequence**	**Retention Time (min)**	**Products**	**Products RMS Mass Error (ppm)**	**Products RMS RT Error (min)**	**Protein ID**
998.5566	1.25	998.5557	8.6193	85	92	(R)ELYLVAYK(T)	31.8514	19	6.1215	0.0102	4360,
931.5101	1.14	931.5095	8.4748	165	172	(R)DNIIDLTK(T)	29.1848	29	14.06	0.0135	4360,
1876.9835	2.35	1876.9626	8.2073	59	74	(R)VFMQHIDVLENSLGFK(F)	40.762	18	13.2013	0.015	4360,
777.3999	1	777.3989	8.195	144	150	(K)DLSSDIK(E)	20.5462	11	23.9939	0.017	4360,
2395.1277	2.14	2395.0977	7.8571	93	113	(K)TPEDGEYFVEYDGGNTFTILK(T)	40.1803	25	28.379	0.028	4360,
1056.5349	2	1056.5255	7.7808	156	164	(K)LCEAHGITR(D)	16.3961	11	18.1757	0.0171	4360,
1303.6945	2	1303.6852	7.7589	165	175	(R)DNIIDLTKTDR(C)	28.6285	12	18.7717	0.0179	4360,
1311.7079	2	1311.6918	7.736	119	128	(R)YVMFHLINFK(N)	40.1412	10	22.3502	0.0224	4360,
1892.9792	2.4	1892.9575	7.5025	59	74	(R)VFMQHIDVLENSLGFK(F)	37.4782	11	19.1344	0.0237	4360,
1327.6982	2.1	1327.6866	7.4515	119	128	(R)YVMFHLINFK(N)	37.2693	9	20.2806	0.0245	4360,
1543.7667	2	1543.7573	6.9647	129	141	(K)NGETFQLMVLYGR(T)	37.9151	6	23.9049	0.0282	4360,
1527.7585	2	1527.7625	6.7206	129	141	(K)NGETFQLMVLYGR(T)	42.7717	5	32.5533	0.0473	4360,
1019.5158	2	1019.5051	6.4729	173	180	(K)TDRCLQAR(G)	31.8685	5	32.5489	0.0311	4360,
406.1961	1	406.1973	0	85	87	(R)ELY(L)	31.83	1	15.8771	0.0058	4360,
600.3441	1	600.3392	0	85	89	(R)ELYLV(A)	31.8428	4	8.4621	0.012	4360,
759.3985	1	759.3883	0	144	150	(K)DLSSDIK(E)	22.1218	0	0	0	4360,
943.4533	2	943.4415	0	157	164	(L)CEAHGITR(D)	16.4003	5	5.7086	0.0144	4360,
913.4875	1	913.4989	0	165	172	(R)DNIIDLTK(T)	31.176	1	38.0372	0.0269	4360,
1285.6631	2	1285.6747	0	165	175	(R)DNIIDLTKTDR(C)	33.5471	3	10.1719	0.0155	4360,
702.4376	1	702.4396	0	167	172	(N)IIDLTK(T)	29.903	2	15.4513	0.0253	4360,
571.3529	1	571.345	0	168	172	(I)IDLTK(T)	28.5373	0	0	0	4360,
961.5411	2	961.5313	0	168	175	(I)IDLTKTDR(C)	28.02	1	23.0307	0.0307	4360,
476.2703	1	476.2715	0	169	172	(I)DLTK(T)	29.9049	1	0.6196	0.0049	4360,
388.1896	1	388.1867	0	85	87	(R)ELY(L)	31.8421	3	33.7737	0.0085	4360,

Spot No.	Accession	Description	mW (Da)	pI (pH)	Peptides	Theoretical Peptides	Coverage (%)	Products	Products RMS Mass Error (ppm)	Products RMS RT Error (min)	Protein ID
10	P02761	Major urinary protein	20723	5.7964	35	20	56.9061	331	16.6652	0.0170851	4360
Precursor MH+ (Da)	z	Peptide MH+ (Da)	Score	Start	End	Sequence	Retention Time (min)	Products	Products RMS Mass Error (ppm)	Products RMS RT Error (min)	Protein ID
1876.9796	2.36	1876.9626	9.191	59	74	(R)VFMQHIDVLENSLGFK(F)	40.2378	43	11.845	0.0182	4360,
1527.7708	1.97	1527.7625	9.0252	129	141	(K)NGETFQLMVLYGR(T)	39.6086	29	12.1723	0.0153	4360,
998.5547	1.33	998.5557	9.0056	85	92	(R)ELYLVAYK(T)	31.5208	18	11.3873	0.0139	4360,
931.5107	1.12	931.5095	8.9457	165	172	(R)DNIIDLTK(T)	28.9851	31	9.8009	0.0159	4360,
2395.117	2.14	2395.0977	8.889	93	113	(K)TPEDGEYFVEYDGGNTFTILK(T)	39.972	34	14.6913	0.0139	4360,
1892.967	2.46	1892.9575	8.8224	59	74	(R)VFMQHIDVLENSLGFK(F)	37.1182	26	16.1742	0.023	4360,
1311.6987	2.01	1311.6918	8.8217	119	128	(R)YVMFHLINFK(N)	39.7858	18	11.3675	0.014	4360,
777.3955	1	777.3989	8.6294	144	150	(K)DLSSDIK(E)	20.4627	16	21.2808	0.0169	4360,
1303.6938	2	1303.6852	8.5054	165	175	(R)DNIIDLTKTDR(C)	28.4951	23	27.3548	0.0159	4360,
1034.539	1.92	1034.5364	8.4102	144	152	(K)DLSSDIKEK(F)	19.7222	8	12.6011	0.0165	4360,
1303.6887	1.98	1303.6852	8.3565	165	175	(R)DNIIDLTKTDR(C)	27.8628	18	26.4502	0.013	4360,
1543.7661	2	1543.7573	8.2158	129	141	(K)NGETFQLMVLYGR(T)	36.0831	14	22.7845	0.018	4360,
1327.6989	2.13	1327.6866	8.1611	119	128	(R)YVMFHLINFK(N)	37.0383	11	13.3657	0.0206	4360,
1549.8035	2	1549.8009	7.9411	34	47	(K)LNGDWFSIVVASNK(R)	41.4496	9	22.9153	0.0186	4360,
1705.9177	2.45	1705.902	7.2158	34	48	(K)LNGDWFSIVVASNKR(E)	38.9028	4	12.0625	0.0181	4360,
1083.5597	2	1083.5656	0	59	67	(R)VFMQHIDVL(E)	41.0598	1	0	0	4360,
776.3893	1	776.3937	0	68	74	(L)ENSLGFK(F)	41.0121	1	25.404	0.0319	4360,
388.1868	1	388.1867	0	85	87	(R)ELY(L)	31.5086	3	14.8704	0.01	4360,
406.1971	1	406.1973	0	85	87	(R)ELY(L)	31.5134	1	0.0789	9.00E-04	4360,
501.2717	1	501.2708	0	85	88	(R)ELYL(V)	31.5091	0	0	0	4360,
600.3402	1	600.3392	0	85	89	(R)ELYLV(A)	31.5077	0	0	0	4360,
593.3662	1	593.3657	0	88	92	(Y)LVAYK(T)	31.5186	1	4.9758	0.0172	4360,
480.2822	1	480.2817	0	89	92	(L)VAYK(T)	31.5109	4	19.5568	0.0098	4360,
381.2138	1	381.2132	0	90	92	(V)AYK(T)	31.5081	1	5.229	0.002	4360,
634.3908	1	634.3923	0	124	128	(H)LINFK(N)	39.7744	2	4.908	0.0231	4360,
521.315	1	521.3082	0	125	128	(L)INFK(N)	39.771	1	0	0	4360,
759.3937	1	759.3883	0	144	150	(K)DLSSDIK(E)	22.0377	0	0	0	4360,
549.2847	1	549.2879	0	146	150	(L)SSDIK(E)	20.4571	8	13.333	0.0211	4360,
554.2471	1	554.2457	0	165	169	(R)DNIID(L)	28.9871	1	0	0	4360,
684.3626	1	684.3563	0	165	170	(R)DNIIDL(T)	29.0077	1	46.3171	0.0072	4360,
702.4363	1	702.4396	0	167	172	(N)IIDLTK(T)	30.2394	2	4.5717	0.018	4360,
589.3573	1	589.3556	0	168	172	(I)IDLTK(T)	28.9858	2	10.1008	0.0312	4360,
458.2605	1	458.2609	0	169	172	(I)DLTK(T)	24.5677	0	0	0	4360,
476.2693	1	476.2715	0	169	172	(I)DLTK(T)	28.9856	0	0	0	4360,
970.468	1	970.4815	0	59	66	(R)VFMQHIDV(L)	41.9823	0	0	0	4360,

Spot No.	Accession	Description	mW (Da)	pI (pH)	Peptides	Theoretical Peptides	Coverage (%)	Products	Products RMS Mass Error (ppm)	Products RMS RT Error (min)	Protein ID
11	P02761	Major urinary protein	20723	5.7964	25	20	61.8785	247	19.335	0.0193695	4360
Precursor MH+ (Da)	z	Peptide MH+ (Da)	Score	Start	End	Sequence	Retention Time (min)	Products	Products RMS Mass Error (ppm)	Products RMS RT Error (min)	Protein ID
998.5544	1.44	998.5557	8.5483	85	92	(R)ELYLVAYK(T)	31.498	18	10.1703	0.0145	4360,
1876.9836	2.41	1876.9626	8.5014	59	74	(R)VFMQHIDVLENSLGFK(F)	40.3035	31	16.1544	0.0205	4360,
931.5101	1.16	931.5095	8.4639	165	172	(R)DNIIDLTK(T)	28.9586	25	14.9228	0.0095	4360,
1527.7745	1.98	1527.7625	8.2166	129	141	(K)NGETFQLMVLYGR(T)	39.5197	20	18.509	0.0216	4360,
1543.7715	2	1543.7573	8.1859	129	141	(K)NGETFQLMVLYGR(T)	35.9962	19	25.447	0.021	4360,
1892.9772	2.47	1892.9575	8.1723	59	74	(R)VFMQHIDVLENSLGFK(F)	36.9987	22	15.5769	0.0219	4360,
1327.6993	2.12	1327.6866	7.8713	119	128	(R)YVMFHLINFK(N)	36.8541	13	22.0713	0.0182	4360,
2395.125	3	2395.0977	7.8371	93	113	(K)TPEDGEYFVEYDGGNTFTILK(T)	39.8091	29	22.5599	0.0219	4360,
1056.533	2	1056.5255	7.5625	156	164	(K)LCEAHGITR(D)	16.1401	7	11.8388	0.0212	4360,
1303.6788	2	1303.6852	7.4582	165	175	(R)DNIIDLTKTDR(C)	27.6828	10	16.1432	0.0257	4360,
1311.7018	2.02	1311.6918	7.4559	119	128	(R)YVMFHLINFK(N)	39.7339	8	37.6777	0.0217	4360,
1303.6947	2	1303.6852	7.4196	165	175	(R)DNIIDLTKTDR(C)	28.409	13	23.0168	0.0162	4360,
1192.565	2	1192.5627	7.182	49	58	(R)EKIEENGSMR(V)	16.0566	7	18.9882	0.0225	4360,
1549.8136	2	1549.8009	6.8339	34	47	(K)LNGDWFSIVVASNK(R)	41.3209	3	20.3089	0.0202	4360,
665.356	1	665.3617	0	69	74	(E)NSLGFK(F)	40.3069	1	23.4188	0.0031	4360,
388.1881	1	388.1867	0	85	87	(R)ELY(L)	31.5135	1	0	0	4360,
406.2002	1	406.1973	0	85	87	(R)ELY(L)	31.5018	1	0.8821	0.0101	4360,
501.2754	1	501.2708	0	85	88	(R)ELYL(V)	31.5116	3	1.875	0.0144	4360,
980.5509	2	980.5451	0	85	92	(R)ELYLVAYK(T)	31.5094	0	0	0	4360,
756.4307	1.21	756.4291	0	87	92	(L)YLVAYK(T)	31.5137	6	7.244	0.0071	4360,
480.2829	1	480.2817	0	89	92	(L)VAYK(T)	31.5115	4	4.1864	0.0073	4360,
1526.746	2	1526.7308	0	129	141	(K)NGETFQLMVLYGR(T)	36.5647	0	0	0	4360,
943.4455	2	943.4415	0	157	164	(L)CEAHGITR(D)	16.132	5	4.8394	0.0279	4360,
684.3615	1	684.3563	0	165	170	(R)DNIIDL(T)	28.9053	1	0	0	4360,
1083.5654	2	1083.5656	0	59	67	(R)VFMQHIDVL(E)	40.2948	0	0	0	4360,

Spot No.	Accession	Description	mW (Da)	pI (pH)	Peptides	Theoretical Peptides	Coverage (%)	Products	Products RMS Mass Error (ppm)	Products RMS RT Error (min)	Protein ID
12	Q6AY80	Ribosyldihydronicotinamide dehydr	26258	7.1646	3	20	17.316	32	31.3643	0.0161382	4698
Precursor MH+ (Da)	z	Peptide MH+ (Da)	Score	Start	End	Sequence	Retention Time (min)	Products	Products RMS Mass Error (ppm)	Products RMS RT Error (min)	Protein ID
2265.0442	2.74	2265.0435	6.3589	214	231	(K)SIWKEEPIHCTPSWYFQG(-)	33.3694	25	29.8045	0.0173	4698,
2510.1426	2.76	2510.206	5.3534	120	141	(R)VLCQGFAFDVPGFYDSGFLKDK(L)	32.1066	7	36.3933	0.0109	4698,
515.2987	1	515.2976	0	214	217	(K)SIWK(E)	36.0314	0	0	0	4698,

Spot No.	Accession	Description	mW (Da)	pI (pH)	Peptides	Theoretical Peptides	Coverage (%)	Products	Products RMS Mass Error (ppm)	Products RMS RT Error (min)	Protein ID
13	P70709	Eosinophil cationic protein	17994	9.8188	4	8	25.1613	44	30.5455	0.024018	2107
Precursor MH+ (Da)	z	Peptide MH+ (Da)	Score	Start	End	Sequence	Retention Time (min)	Products	Products RMS Mass Error (ppm)	Products RMS RT Error (min)	Protein ID
2194.0977	2.98	2194.071	6.3014	63	82	(K)DINTFLHTSFASVVGVCGNR(N)	33.2235	32	33.2235	0.0194	2107,
2301.1592	2.71	2301.148	5.7709	101	119	(R)VSITFCNLTTPARIYTQCR(Y)	35.2071	10	35.2071	0.0342	2107,
761.3809	1	761.3723	0	76	82	(V)VGVCGNR(N)	33.4329	1	33.4329	0.0059	2107,
591.282	1	591.2773	0	63	67	(K)DINTF(L)	31.857	1	31.857	0	2107,

Spot No.	Accession	Description	mW (Da)	pI (pH)	Peptides	Theoretical Peptides	Coverage (%)	Products	Products RMS Mass Error (ppm)	Products RMS RT Error (min)	Protein ID
14	G3V664	Protein LOC100912262	102644	6.0073	5	86	9.772	52	28.2126	0.0201711	21148
Precursor MH+ (Da)	z	Peptide MH+ (Da)	Score	Start	End	Sequence	Retention Time (min)	Products	Products RMS Mass Error (ppm)	Products RMS RT Error (min)	Protein ID
1120.5688	2	1120.5634	6.1927	692	700	(R)EQVQDLFNK(K)	33.219	18	20.5698	0.0134	21148,
3264.5625	2.93	3264.5664	6.0326	239	268	(K)VSPQDLTPTATPSSMANFLYSTSMPNHTIR(E)	39.3449	15	26.783	0.0202	21148,
3356.75	4	3356.7646	5.2826	594	622	(K)FLMHPEELFVLGLPEGISLRRPNCFGIAK(L)	40.9815	6	33.1685	0.0306	21148,
1109.5656	2	1109.566	4.9876	568	576	(K)QVEMLFNTK(Y)	25.2417	4	40.4486	0.025	21148,
1417.6621	2	1417.6859	4.7232	90	102	(R)GPLWNDPEAGHPK(K)	30.6563	9	33.1127	0.0202	21148,

Spot No.	Accession	Description	mW (Da)	pI (pH)	Peptides	Theoretical Peptides	Coverage (%)	Products	Products RMS Mass Error (ppm)	Products RMS RT Error (min)	Protein ID
15	F1M6M6	Protein 1700008I05Rik Fragment	47980	5.3818	2	27	16.5877	18	28.8367	0.0329475	18599
Precursor MH+ (Da)	z	Peptide MH+ (Da)	Score	Start	End	Sequence	Retention Time (min)	Products	Products RMS Mass Error (ppm)	Products RMS RT Error (min)	Protein ID
4534.2886	4.52	4534.2417	5.9835	67	106	(K)QETEHGALDVPHLSNYILNLMALMCAPVRDEAVQELESIK(D)	41.7321	9	29.8486	0.04	18599,
3347.698	3.39	3347.631	5.4183	284	313	(K)ALTEEFSSKPEESMLSVSEQVSQEIHQGLK(E)	41.3269	9	27.788	0.024	18599,

Spot No.	Accession	Description	mW (Da)	pI (pH)	Peptides	Theoretical Peptides	Coverage (%)	Products	Products RMS Mass Error (ppm)	Products RMS RT Error (min)	Protein ID
16	Q5XI73	Rho GDP dissociation inhibitor 1	23392	4.916	2	18	12.7451	11	14.0749	0.0263902	2684
Precursor MH+ (Da)	z	Peptide MH+ (Da)	Score	Start	End	Sequence	Retention Time (min)	Products	Products RMS Mass Error (ppm)	Products RMS RT Error (min)	Protein ID
1650.9299	2	1650.9174	6.3409	59	74	(R)VAVSADPNVPNVIVTR(L)	30.4575	8	12.4471	0.0215	2684,
1202.6156	2	1202.6263	5.4358	34	43	(K)SIQEIQELDK(D)	28.3901	3	17.6982	0.0364	2684,

Spot No.	Accession	Description	mW (Da)	pI (pH)	Peptides	Theoretical Peptides	Coverage (%)	Products	Products RMS Mass Error (ppm)	Products RMS RT Error (min)	Protein ID
17	Q8VI32	Activated B cell RT1Bl alpha chain	28456	4.6201	2	13	20.7031	17	31.7452	0.0260852	10173
Precursor MH+ (Da)	z	Peptide MH+ (Da)	Score	Start	End	Sequence	Retention Time (min)	Products	Products RMS Mass Error (ppm)	Products RMS RT Error (min)	Protein ID
2720.292	3.41	2720.2952	6.0183	151	174	(R)NSKPVTEGVYETSFLSNPDHSFHK(M)	32.8306	12	29.3519	0.0246	10173,
3336.788	4.59	3336.8176	5.1147	122	150	(K)SPVLLGQPNTLICFVDNIFPPVINITWLR(N)	38.9994	5	36.8605	0.0294	10173,

Spot No.	Accession	Description	mW (Da)	pI (pH)	Peptides	Theoretical Peptides	Coverage (%)	Products	Products RMS Mass Error (ppm)	Products RMS RT Error (min)	Protein ID
18	Q68J51	AC1147 Fragment	9924	6.9258	3	7	53.6842	17	30.271	0.0278817	33348
Precursor MH+ (Da)	z	Peptide MH+ (Da)	Score	Start	End	Sequence	Retention Time (min)	Products	Products RMS Mass Error (ppm)	Products RMS RT Error (min)	Protein ID
2926.3955	2.88	2926.367	6.0852	1	27	(-)LGGAICLVNSDNERDSGMLTSYATPER(S)	38.3706	10	27.2441	0.0331	33348,
2370.1812	3	2370.187	5.3129	62	85	(R)ATLTGSACAYPTPAGAGNPLNPIR(D)	36.0684	6	27.4509	0.0176	33348,
2068.0176	2	2067.9917	0	65	85	(L)TGSACAYPTPAGAGNPLNPIR(D)	35.671	1	0	0	33348,

Spot No.	Accession	Description	mW (Da)	pI (pH)	Peptides	Theoretical Peptides	Coverage (%)	Products	Products RMS Mass Error (ppm)	Products RMS RT Error (min)	Protein ID
19	D4AEG9	Protein Hesx1	21559	8.9941	3	23	19.4595	22	22.6842	0.0243555	8548
Precursor MH+ (Da)	z	Peptide MH+ (Da)	Score	Start	End	Sequence	Retention Time (min)	Products	Products RMS Mass Error (ppm)	Products RMS RT Error (min)	Protein ID
703.3593	1	703.3621	6.5145	139	144	(R)EDLAQK(L)	18.5883	10	15.3808	0.0201	8548,
2222.1177	3	2222.101	5.4719	13	32	(R)ESKPSPCSFSIESILGLDQK(K)	34.3852	7	22.6973	0.0309	8548,
1922.9332	2	1922.9099	4.8814	129	144	(R)MNCYPGIDIREDLAQK(L)	35.9174	5	32.707	0.0215	8548,

Spot No.	Accession	Description	mW (Da)	pI (pH)	Peptides	Theoretical Peptides	Coverage (%)	Products	Products RMS Mass Error (ppm)	Products RMS RT Error (min)	Protein ID
20	A0JN29	Limb and neural patterns	42143	9.5654	6	33	13.8298	30	24.9611	0.0289515	15279
Precursor MH+ (Da)	z	Peptide MH+ (Da)	Score	Start	End	Sequence	Retention Time (min)	Products	Products RMS Mass Error (ppm)	Products RMS RT Error (min)	Protein ID
824.4976	2	824.4989	6.6714	211	218	(R)TVAPALPR(R)	19.0996	15	19.8665	0.0244	15279,
4588.21	4.37	4588.189	5.2609	321	364	(R)QAVEGSSSTGPMLLESVPSPENQLTEGGTIPQMSHFLSEISGMK(S)	44.175	5	24.2104	0.0322	15279,
440.2492	1	440.2504	0	211	215	(R)TVAPA(L)	19.1105	4	25.3374	0.0331	15279,
535.3277	1	535.3239	0	211	216	(R)TVAPAL(P)	19.0858	2	0	0	15279,
385.2566	1	385.2558	0	216	218	(A)LPR(R)	19.1142	1	60.1873	0.0226	15279,
422.2434	1	422.2398	0	211	215	(R)TVAPA(L)	19.1043	3	24.96	0.0402	15279,

Spot No.	Accession	Description	mW (Da)	pI (pH)	Peptides	Theoretical Peptides	Coverage (%)	Products	Products RMS Mass Error (ppm)	Products RMS RT Error (min)	Protein ID
21	O08773	Regulator of G protein signaling 14	59454	7.2012	8	46	10.6618	32	32.6215	0.0296785	5790
Precursor MH+ (Da)	z	Peptide MH+ (Da)	Score	Start	End	Sequence	Retention Time (min)	Products	Products RMS Mass Error (ppm)	Products RMS RT Error (min)	Protein ID
842.5085	1	842.4982	6.5297	334	341	(R)GLSLPDIK(V)	19.2757	12	16.8386	0.0229	5790,
2940.3835	3.26	2940.4333	5.3153	257	284	(R)ESQGSLNSSASLDLGFLAFVSSKSESHR(K)	44.6571	8	39.8968	0.0349	5790,
2171.0417	2	2171.0461	5.1777	470	491	(K)DSHLPPLSSSLSVEDASGSTGK(R)	45.2022	5	48.0239	0.0217	5790,
371.2266	1	371.2289	0	334	337	(R)GLSL(P)	21.675	2	15.1658	0.0563	5790,
468.2841	1	468.2817	0	334	338	(R)GLSLP(D)	20.1281	0	0	0	5790,
824.5002	2	824.4876	0	334	341	(R)GLSLPDIK(V)	21.1	0	0	0	5790,
672.4017	1	672.3927	0	336	341	(L)SLPDIK(V)	20.6449	2	48.7889	0.0244	5790,
353.2181	1	353.2183	0	334	337	(R)GLSL(P)	19.272	3	19.7023	0.0274	5790,

Spot No.	Accession	Description	mW (Da)	pI (pH)	Peptides	Theoretical Peptides	Coverage (%)	Products	Products RMS Mass Error (ppm)	Products RMS RT Error (min)	Protein ID
22	E9PSX4	Protein Il3ra	41979	5.9985	5	28	26.4249	26	30.4443	0.0239638	18709
Precursor MH+ (Da)	z	Peptide MH+ (Da)	Score	Start	End	Sequence	Retention Time (min)	Products	Products RMS Mass Error (ppm)	Products RMS RT Error (min)	Protein ID
827.4403	1.98	827.437	6.437	56	64	(R)AEGGALGPR(F)	20.207	13	33.2827	0.0157	18709,
2314.2007	2.78	2314.1714	5.2724	252	271	(R)FQTAFVFTLQINQSSQTEPK(L)	34.932	5	23.8378	0.02	18709,
5547.7207	4	5547.8037	5.1443	294	346	(K)ATALDSGVESDWSKAWSLDCGPTATLATPMTSLLLAGAGAVLTVMAVLLLCWR(K)	45.34	3	16.9243	0.0406	18709,
1219.6709	2	1219.5854	4.918	45	55	(R)LTWEWGGGGTR(A)	31.7029	3	31.4679	0.0244	18709,
1013.5172	2	1013.5374	4.6478	35	43	(R)NLSIDPAQR(R)	24.3697	2	38.9005	0.0387	18709,

Spot No.	Accession	Description	mW (Da)	pI (pH)	Peptides	Theoretical Peptides	Coverage (%)	Products	Products RMS Mass Error (ppm)	Products RMS RT Error (min)	Protein ID
23	F1LP66	Mitogen activated protein kinase 8	47129	7.2935	3	37	14.0777	27	25.1214	0.0296946	23854
Precursor MH+ (Da)	z	Peptide MH+ (Da)	Score	Start	End	Sequence	Retention Time (min)	Products	Products RMS Mass Error (ppm)	Products RMS RT Error (min)	Protein ID
787.4777	2	787.4785	5.8916	368	374	(R)TKNGVIR(G)	18.9854	6	20.7343	0.0405	23854,
3726.6934	3	3726.6858	5.3835	375	412	(R)GQPSPLGAAVINGSQHPSSSPSVNDMSSMSTDPTLASD(-)	39.6455	12	30.8244	0.0245	23854,
1712.835	2	1712.8239	4.9358	342	354	(K)QLDEREHTIEEWK(E)	32.4058	9	18.4335	0.0275	23854,

Spot No.	Accession	Description	mW (Da)	pI (pH)	Peptides	Theoretical Peptides	Coverage (%)	Products	Products RMS Mass Error (ppm)	Products RMS RT Error (min)	Protein ID
24	D4A644	Protein Map7d1	90363	10.4678	5	78	5.1408	25	32.7837	0.0295882	11060
Precursor MH+ (Da)	z	Peptide MH+ (Da)	Score	Start	End	Sequence	Retention Time (min)	Products	Products RMS Mass Error (ppm)	Products RMS RT Error (min)	Protein ID
743.4438	1.01	743.441	6.3244	315	321	(R)SAVTLPR(N)	23.6393	11	18.8274	0.0314	11060,
1129.4949	1	1129.5009	4.9896	484	494	(K)ESPSPSGPEDK(N)	35.8699	4	41.5911	0.0222	11060,
1713.8219	2	1713.829	4.7453	114	131	(R)SSQPSPTAVPASDSPSAK(Q)	33.7492	5	35.3983	0.0274	11060,
688.3597	1	688.3624	4.6895	196	201	(R)AALEER(Q)	28.5215	3	39.3594	0.0345	11060,
385.256	1	385.2558	0	319	321	(T)LPR(N)	23.6351	2	50.6861	0.0295	11060,
